# Next generation risk assessment of human exposure to estrogens using safe comparator compound values based on in vitro bioactivity assays

**DOI:** 10.1007/s00204-023-03480-w

**Published:** 2023-04-22

**Authors:** Tessa C. A. van Tongeren, Si Wang, Paul L. Carmichael, Ivonne M. C. M. Rietjens, Hequn Li

**Affiliations:** 1 Division of Toxicology, Wageningen University and Research, 6700 EA Wageningen, The Netherlands; 2 Unilever Safety and Environmental Assurance Centre, Sharnbrook, Bedfordshire, MK44 1LQ UK

**Keywords:** Risk assessment, 3R compliant method, Estrogen receptor, Dietary Comparator, In vitro/in silico approaches

## Abstract

In next generation risk assessment (NGRA), the Dietary Comparator Ratio (DCR) can be used to assess the safety of chemical exposures to humans in a 3R compliant approach. The DCR compares the Exposure Activity Ratio (EAR) for exposure to a compound of interest (EAR_test_) to the EAR for an established safe exposure level to a comparator compound (EAR_comparator_), acting by the same mode of action. It can be concluded that the exposure to a test compound is safe at a corresponding DCR ≤ 1. In this study, genistein (GEN) was selected as a comparator compound by comparison of reported safe internal exposures to GEN to its BMCL_05_, as no effect level, the latter determined in the in vitro estrogenic MCF7/Bos proliferation, T47D ER-CALUX, and U2OS ERα-CALUX assay. The EAR_comparator_ was defined using the BMCL_05_ and EC_50_ values from the 3 in vitro assays and subsequently used to calculate the DCRs for exposures to 14 test compounds, predicting the (absence of) estrogenicity. The predictions were evaluated by comparison to reported in vivo estrogenicity in humans for these exposures. The results obtained support in the DCR approach as an important animal-free new approach methodology (NAM) in NGRA and show how in vitro assays can be used to define DCR values.

**Supplementary Information:**

The online version contains supplementary material available at 10.1007/s00204-023-03480-w.

## Introduction

The use of animal testing for toxicological risk assessment is under debate because of ethical, economic, and legislative issues, and their adequacy to accurately represent the human situation. In contrast, in next generation risk assessment (NGRA), in silico and in vitro approaches are used to assure human safety (Becker et al. 2015; Dent et al. 2019). The Dietary Comparator Ratio (DCR) is an NGRA compliant tool (Becker et al. 2015) which compares the Exposure Activity Ratio (EAR) for exposure to a compound of interest (EAR_test_) to the EAR for an established safe level of human exposure to a comparator compound (EAR_comparator_), acting by the same mode of action. In the EAR, the unbound internal concentration of a compound at a defined exposure level is divided by its in silico or in vitro derived half maximum effective concentration (EC_50_) (Becker et al. 2015). A DCR ≤ 1 for the compound of interest, calculated as the ratio EAR_test_/EAR_comparator_, indicates that the respective exposure scenario will be safe.

**Fig. 1:**
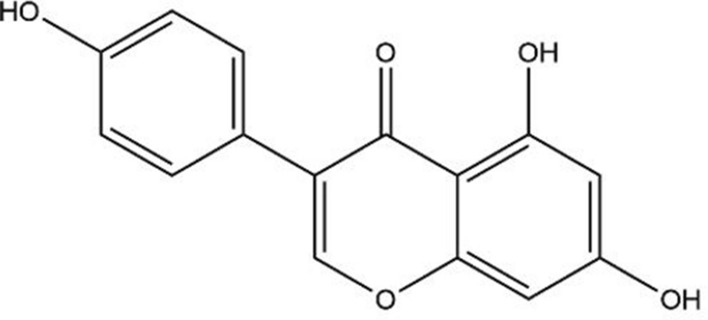
Structure formula of genistein (GEN)

Proof of principle for the DCR approach (evaluating the safety of exposure scenarios to estrogenic and anti-androgenic compounds) was originally reported by Becker et al. (2015) and Dent et al. (2019). Becker et al. (2015) defined the EAR_comparator_ based on reported human exposures to the phytoestrogen (isoflavone) genistein (GEN, Fig. 1), mostly found in soybeans (Elsenbrand 2007), from different diets. In this study it was indicated that these dietary exposure levels were considered conservative and health protective in humans. Results obtained indicated that 6 out of the 30 exposure scenarios to several test compounds had a DCR > 1 and the authors concluded that these exposures should be prioritized for safety assessment (Becker et al. 2015). However, no evaluation against information on corresponding in vivo estrogenic activity at these exposure scenarios was made to further affirm this prioritization. Dent et al. (2019) defined the EAR_comparator_ for anti-androgenic effects based on diindolylmethane (DIM) from the intake of 50 g Brussels sprouts with a history of safe use. Whilst protective, this comparator exposure scenario appeared to be overly conservative since all exposure scenarios to the test compounds had a DCR > 1, including exposures with supportive data on the absence of corresponding in vivo anti-androgenic effects in humans. Previously, we reported a newly defined EAR_comparator_ based on safe levels of exposure to anti-androgens which was solely based on in vitro data. It was proven that this EAR_comparator_ was adequately protective for evaluating the safety of exposure scenarios to anti-androgenic compounds in the DCR approach (van Tongeren et al. 2021).

The aim of the current study was to define and use new EAR_comparator_ values based on safe levels of exposure to estrogens solely based on in vitro data to evaluate human exposures to estrogens. These newly defined EAR_comparator_ values were based on the in vitro MCF-7/Bos proliferation assay, T47D estrogen receptor (ER)-CALUX assay, and U2OS ERα-CALUX assay using GEN as comparator compound. A series of biologically relevant exposure scenarios to 14 compounds constituting endogenous hormones, phthalates, ethyl paraben, pesticides, bisphenol A, phytoestrogens, the mycotoxin zearalenone, and drugs with information regarding accompanying in vivo estrogenic activity were included, generating EAR_test_ values for exposure scenarios that were known to be positive or negative for estrogenic effects, or in some cases still unknown. This enabled evaluation of the corresponding DCR values obtained when using the newly defined EAR_comparator_ values.

## Methods

### Workflow of the DCR approach

**Fig. 2:**
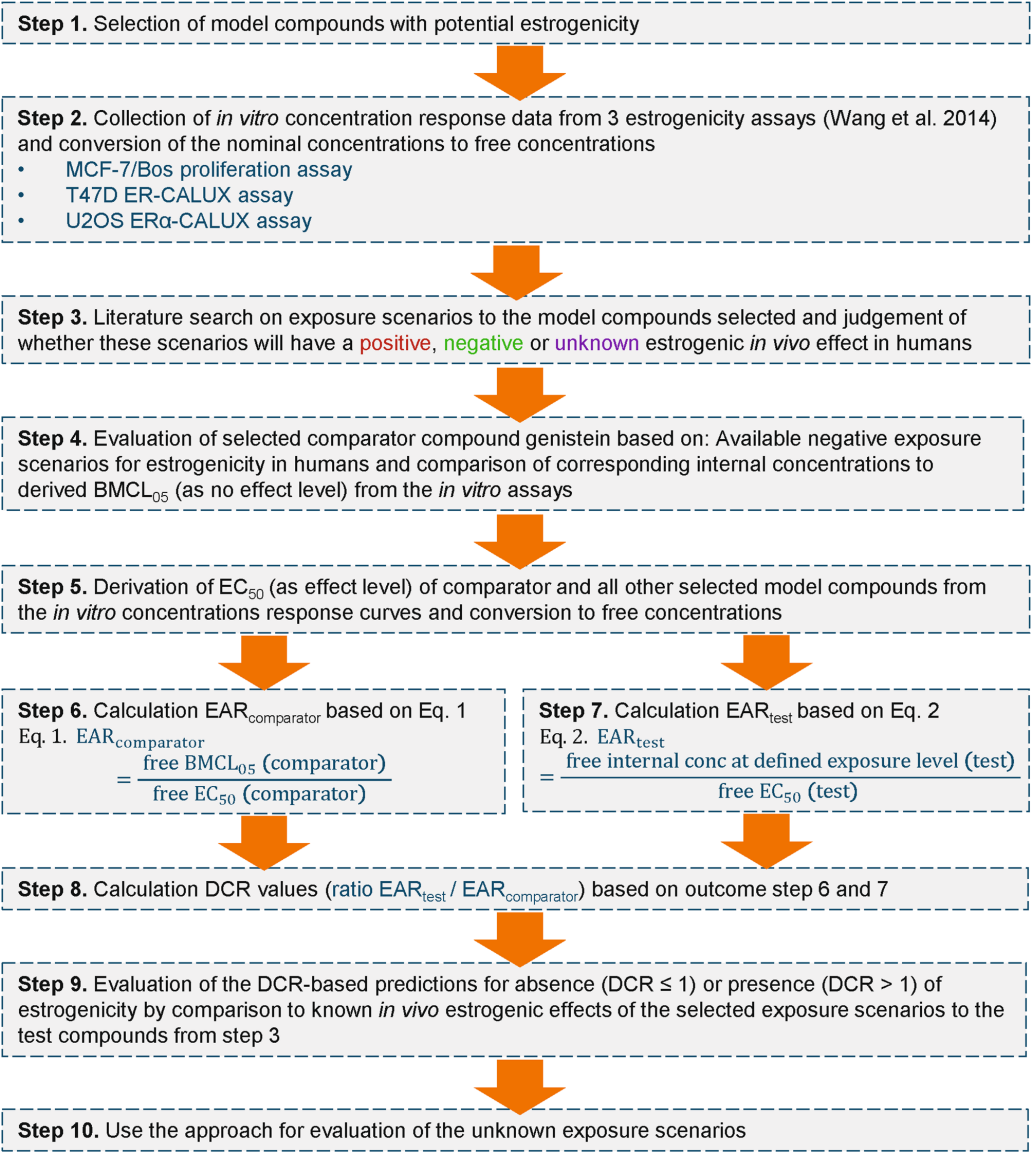
Schematic scheme of the workflow used in the present study executing the DCR approach to evaluate exposure scenarios to (putative) estrogenic compounds using data from in vitro bioassays

The DCR approach was executed following multiple steps which are depicted in the workflow (Fig. 2).

Step 1: Selection of model compounds with potential estrogenicity.

Compounds that were active in the in vitro estrogenic MCF-7/Bos proliferation assay, T47D ER-CALUX assay, or U2OS ERα-CALUX assay were selected as model compounds. For these compounds in vitro concentration-response data and in vivo estrogenicity data for selected exposure regimens in humans were collected in Step 2 and 3, respectively. From these compounds a comparator compound was selected in Step 4.

Step 2: Collection of in vitro concentration-response data from 3 estrogenicity assays.

The concentration-response data of the selected model compounds in the in vitro estrogenic MCF-7/Bos proliferation assay, T47D ER-CALUX assay, or U2OS ERα-CALUX assay were derived from Wang et al. (2014). In short, the human breast cancer estrogenic-sensitive MCF-7 cells were exposed to concentration ranges of the compounds for 6 days in the MCF-7/Bos proliferation assay. The number of cells was measured with the Burton diphenylamine assay, quantifying the amount of DNA per well. In the CALUX assays, the human breast carcinoma T47D cells endogenously expressing the ERα and ERβ and the human osteosarcoma U2OS cells transfected with the ERα were exposed to increasing concentrations of the compounds for 24 h whereafter the luciferase reporter gene activity as the fold ER induction was measured. The concentrations were converted to the free concentrations using the fraction unbound in vitro (f_ub in vitro_) since only the free unbound form is assumed to induce toxicity. This f_ub in vitro_ and also the fraction unbound in vivo (f_ub in vivo_) of the model compounds were determined as described by van Tongeren et al. (2021). In short, the f_ub in vivo_ values were calculated using the ADMET predictor™ version 9.6 (Simulation Plus Inc.). The f_ub in vitro_ at the 5% protein content present in the in vitro media (Wang et al. 2014) was linear extrapolated based on an f_ub in vitro_ = 1.0 at 0% protein and the f_ub in vivo_ values at an 8% protein content in human plasma (Mescher 2009; Mathew et al. 2020). In line with literature data, it was assumed that the protein content and fraction unbound are linearly related (Gülden et al. 2002).

Step 3: Literature search on exposure scenarios to the model compounds selected and judgement of whether these scenarios will have a positive, negative or unknown estrogenic in vivo effect in humans.

Human exposure scenarios to the model compounds were gathered from literature to be evaluated in the DCR approach and provided information regarding the in vivo estrogenicity in humans to evaluate the DCR-based predictions. The compounds at the respective dose levels were reported to be positive or negative for in vivo estrogenicity in humans. When information on the in vivo estrogenicity was not reported, a comparison of the corresponding intake level of the test compound to safe reference dose values was made to judge whether the exposure would be positive or negative for in vivo estrogenicity. When no intake levels but only internal exposure levels were reported, it was assumed that at the corresponding external exposure levels the occurrence of in vivo estrogenic effects was unknown. The online database PubMed was used for the literature search. The key words included the compound name AND human AND internal/plasma/in vivo AND exposure/levels/concentrations, the compound name AND human dietary intake, the compound name AND human clinical trial/study, or the compound name AND human pharmacokinetic/biomonitoring (study). Studies reporting quantified plasma, serum or blood concentrations upon exposure to the model compound in humans were included. Serum concentrations were assumed to be equal to plasma concentrations. Blood concentrations were transformed to corresponding plasma concentrations using the ADMET predictor™ predicted blood to plasma ratio (R_b2p_). Furthermore, the units of the reported internal concentrations were transformed to µM using the molecular weight of the respective compound and the concentrations were transformed to the corresponding free concentrations using the ADMET predictor™ predicted f_ub in vivo_.

Step 4: Evaluation of the selected comparator compound genistein based on available negative exposure scenarios and comparison of corresponding internal concentrations to derived BMCL_05_ (no effect level) values from the in vitro assays.

GEN was selected as the comparator compound based on available negative exposure scenarios for estrogenicity in humans (Becker et al. 2015) (Supplementary material S1) and comparison of the corresponding free internal levels to the derived free BMCL_05_ values as no effect levels from the in vitro assays. This comparison was to confirm that the free BMCL_05_ values are below the free plasma concentrations at the selected safe exposure scenarios for the comparator compound genistein and thus will not induce an estrogenic effect.

Step 4a: Derivation of free internal concentrations corresponding to negative exposure scenarios for the comparator compound.

The reported human internal plasma or serum concentrations of GEN resulting from a Western diet, an Asian diet, or GEN supplements (Becker et al. 2015) (Supplementary material S1), were considered to be conservative and not associated with any adverse health effects in humans. A Western diet is an animal sourced diet with an overall high fat and sugar intake and a lower vegetable, fruit, legumes, whole cereals, raw foods, and fibers intake (Adlercreutz 1998; Rizzello et al. 2019). Western dietary intake of GEN amounts to 0.003–0.01 mg/kg body weight (bw)/day (Aguilar et al. 2015). An Asian diet is a plant sourced diet with a high intake of soy and soy based products (Elsenbrand 2007) leading to a GEN intake of 0.21–0.71 mg/kg bw/day (Rietjens et al. 2013). Supplementary intake of GEN amounts to 0.43–13 mg/kg bw/day (Risk Assessment for Peri- and Post-Menopausal Women Taking Food Supplements Containing Isolated Isoflavones 2015). Only plasma levels of unconjugated GEN were used for comparison since the unconjugated form of GEN is known to be active (Hosoda et al. 2011). When the internal concentrations of GEN were reported in the conjugated + unconjugated form, correction with a factor 0.003 was made to obtain the internal concentrations of unconjugated GEN since 0.3% of GEN is reported to exist in the unconjugated form in plasma (Becker et al. 2015).

Step 4b: Derivation of the free BCML_05_ for the comparator compound as no effect level from the 3 in vitro assays.

To derive the no effect level of GEN, a benchmark dose analysis was performed of the in vitro concentration-response data of the 3 in vitro estrogenicity assays to obtain the BMC causing a 5% increase in response compared to the control (BMC_05_) and the upper (BMCU_05_) and lower (BMCL_05_) bound of its 95% confidence interval (EFSA 2017). The derived BMCL_05_ values reflect the concentrations where no biologically significant ER-mediated effects occur since the BMCL_05_ resembles a no observed adverse effect level (EFSA 2017) and thus are considered as the safe internal exposure levels, which can be used to set the EAR_comparator_. The BMC analysis was performed using the BMDS3.2.1 software (U.S. EPA). All models (Exponential, Hill, Power, Linear and Polynomial) were fitted for continuous data for a BMR type Hybrid model-extra risk with normal distribution and constant variance. Acceptance criteria for a dose–response was indicated with a *p* value > 0.01, and a BMDU_05_: BMDL_05_ ratio (precision factor) below 3 while the lowest AIC was used to select the preferred model (US Environmental Protection Agency 2012; EFSA 2017).

Step 4c. Comparison of the free internal concentrations of the non-estrogenic exposures to the comparator to its free BCML_05_.

The derived free in vitro BMCL_05_ values of GEN were used as surrogate for the free internal concentrations and considered equal to the free in vivo BMCL_05_. This enables comparison to the free internal concentrations of the non-estrogenic exposure scenarios to GEN to evaluate whether the BMCL_05_ can indeed be considered to reflect a safe exposure scenario so that it can be used to define the EAR_comparator_.

Step 5: Derivation of EC_50_ values (as effect levels) from the in vitro concentration-response curves and conversion to free concentrations.

The EC_50_ values from the concentration-response data of the 3 in vitro estrogenicity assays (Wang et al. 2014), were converted to free EC_50_ values to be used as the effect levels of the comparator and test compounds. The free EC_50_ of GEN was used to calculate the EAR_comparator_ in Step 6 whereas those of all other selected model compounds were used to calculate the EAR_test_ in Step 7.

Step 6: Calculation of the EAR_comparator_ values.

With the free BMCL_05_ and free EC_50_ values of the comparator compound GEN derived from the in vitro estrogenic MCF-7/Bos proliferation assay, T47D ER-CALUX assay, and U2OS ERα-CALUX assay, the EAR_comparator_ values were calculated following Eq. 1.

1 $${\mathrm{EAR}}_{\mathrm{comparator}}=\frac{\textrm{ free BMCL}_{05} \left(\mathrm{comparator}\right) }{\textrm{free E} {\mathrm{C}}_{50} \left(\mathrm{comparator}\right)}$$

The free BMCL_05_ and free EC_50_ values of GEN were derived from the in vitro MCF-7/Bos proliferation assay, T47D ER-CALUX assay, or U2OS ERα-CALUX assay (Wang et al. 2014), transforming the nominal concentrations to free concentration using the f_ub in vitro_. The free in vitro BMCL_05_ was considered equal to the free in vivo BMCL_05_ and represents an internal no effect level. It is also of interest to note that the EAR_comparator_ remains unaffected by the correction for protein binding since the correction will affect the nominator and denominator of Eq. 1 in the same way.

Step 7: Calculation of EAR_test_ values.

With the derived free internal concentrations at the respective exposure scenarios from literature of the 14 test compounds and their free EC_50_ values derived from the 3 in vitro estrogenicity assays, the EAR_test_ values were calculated using Eq. 2.

2 $${\mathrm{EAR}}_{\mathrm{test}} =\frac{\mathrm{ free\, internal\, concentration\, at\, defined\, exposure\, level }\left(\mathrm{test}\right) }{{\textrm{free EC}}_{50} \left(\mathrm{test}\right)}$$

The free internal concentration at a defined exposure level of the test compounds was derived from literature reported human in vivo data, which often also included its variability presented as percentiles, range or standard deviation. The corresponding lowest, mean, and highest reported free internal concentrations of the exposure scenarios were selected for this evaluation and the corresponding EAR_test_ values were calculated. This resulted in corresponding lowest, mean, and highest EAR_test_ values. When no distribution was reported, no variability was included resulting in one corresponding EAR_test_ value for the respective exposure scenario. Reported nominal concentrations were transformed to free concentrations using the f_ub in vivo_. The free EC_50_ values were calculated based on the EC_50_ values derived from the concentration-response curves in the MCF-7/Bos proliferation assay, T47D ER-CALUX assay, or U2OS ERα-CALUX assay (Wang et al. 2014), transforming the nominal concentrations to free concentration using the f_ub in vitro_.

Step 8: Calculation of DCR values.

With the obtained EAR_comparator_ and EAR_test_ values, the DCR values were calculated using Eq. 3, generating the DCR values of the test compounds based on each of the 3 in vitro estrogenicity assays using GEN as comparator compound.

3 $$\mathrm{DCR }= \frac{{\mathrm{EAR}}_{\mathrm{test}}}{{\mathrm{EAR}}_{\mathrm{comparator}}}$$

Lowest, mean, and highest DCR values were obtained whenever it was possible in Step 7 to derive from the exposure data of the test compounds lowest, mean, and highest EAR_test_ values. The highest, or when not available the mean, DCR value was used to make a conservative DCR-based safety decision of the respective exposure scenario to the test compound. A DCR ≤ 1 indicates that the corresponding exposure scenario to the test compound will unlikely induce estrogenicity whereas a DCR > 1 indicates the opposite.

Step 9: Evaluation of the DCR-based predictions of the selected exposure scenarios.

To evaluate the DCR outcomes, a comparison was made between the obtained DCR values and actual knowledge on the in vivo estrogenic effects at the corresponding exposure scenario for the test compounds in humans as taken from literature in Step 3. When the exposure scenario was reported to be negative or positive for estrogenicity, a DCR ≤ 1 or > 1 is expected, respectively.

Step 10: Use the approach for evaluation of the unknown exposure scenarios.

After evaluation of the DCR-based predictions of the exposures being negative or positive for estrogenicity, DCR-based predictions were made to evaluate the safety of the exposure scenarios to the test compounds for which it was unknown whether or not they would result in in vivo estrogenicity in humans.

## Results

Step 1: Selection of model compounds with potential estrogenicity.

15 compounds including endogenous hormones, phthalates, ethyl paraben, pesticides, bisphenol A, phytoestrogens, the mycotoxin zearalenone, and drugs were active in the in vitro estrogenic MCF-7/Bos proliferation assay, T47D ER-CALUX assay, or U2OS ERα-CALUX assay and were included as model compounds (Table 1).

**Table 1:** Th﻿e 15 model compounds selected in this study that were observed to have estrogenic activity in the MCF-7/Bos proliferation assay, T47D ER-CALUX assay, or U2OS ERα-CALUX assay (Wang et al. 2014)

Compound group	Test compounds
Endogenous hormones	17β-Estradiol (E2)
	Testosterone (T)
Phthalates	Butylbenzyl phthalate (BBzP)
	Di-n-butyl phthalate (DBP)
Paraben	Ethyl paraben (EP)
Pesticides	Kepone (KEP)
	o,p’-Dichlorodiphenyltrichloroethane (DDT)
Bisphenol	Bisphenol A (BPA)
Phytoestrogens	Genistein (GEN)
	Coumestrol (COU)
	Apigenin (API)
Mycotoxin	Zearalenone (ZEA)
Drugs	17α-Ethinyl estradiol (EE)
	Diethylstilbestrol (DES)
	Tamoxifen (TAM)

Step 2: Collection of in vitro concentration-response data from 3 estrogenicity assays.

The in vitro concentration-response data of the selected model compounds from the MCF-7/Bos proliferation assay, T47D ER-CALUX assay, and U2OS ERα-CALUX assay were taken as reported by Wang et al. (2014). The concentrations were converted to free concentrations using the f_ub in vitro_. The f_ub in vitro_ and f_ub in vivo_ values of the model compounds are listed in Table 2. The f_ub in vivo_ values were predicted with the ADMET predictor™. The f_ub in vitro_ values at a 5% protein content in the in vitro media were linear extrapolated based on the f_ub in vivo_ at an 8% human plasma protein content, setting the f_ub_ at 1.0 in the absence of protein (van Tongeren et al. 2021).

**Table 2:** The ADMET predictor™ predicted f_ub in vivo_ values and the linear extrapolated f_ub in vitro_ values of the model compounds

Compound	f_ub in vivo_	f_ub in vitro_
GEN	0.07	0.42
E2	0.08	0.42
T	0.16	0.48
BBzP	0.04	0.40
DBP	0.06	0.41
EP	0.20	0.50
O,p’-DDT	0.03	0.39
KEP	0.08	0.43
BPA	0.09	0.43
API	0.06	0.41
COU	0.08	0.43
ZEA	0.07	0.42
EE	0.05	0.41
TAM	0.04	0.40
DES	0.04	0.40

Step 3: Literature search on exposure scenarios to the model compounds selected and judgement of whether these scenarios will have a positive, negative or unknown estrogenic in vivo effect in humans.

Literature reported exposure scenarios for the 15 model compounds with

information regarding accompanying in vivo estrogenic effects in humans were collected. 21 Reports on exposures to GEN were available which were indicated to be conservative and health protective in humans (Becker et al. 2015) (Supplementary material S1) and thus considered negative for in vivo estrogenicity. For the remaining compounds, the reported internal concentrations and corresponding free internal concentrations of the corresponding exposure scenarios are compiled in Table 3. In Table 4, the evaluation of the likely occurrence of in vivo estrogenic effects at the exposure scenarios for these model compounds is summarized. This evaluation was based on reports of in vivo estrogenic effects at the dose levels applied or comparison of the reported intake levels to safe reference values like acceptable daily intakes (ADIs). The outcomes were used as the basis to label the exposure as positive or negative for in vivo estrogenicity. Based on the information on the exposure scenarios and the (clinical) data on accompanying in vivo estrogenic effects, 7 of the 41 evaluated exposure scenarios were labelled to be negative and 8 to be positive for in vivo estrogenicity (Table 4). From comparison of reported exposure levels to safe reference values for the model compounds, 8 of the 41 evaluated exposure scenarios were indicated to be negative and 7 to be positive for in vivo estrogenicity. For 11 exposure scenarios the corresponding in vivo estrogenicity was not reported, no dose levels were provided or no safe reference levels were available and therefore the in vivo estrogenic effects induced by the corresponding exposures was listed as unknown (Table 4).

**Table 3:** Literature reported exposure scenarios to the model compound with the corresponding nominal internal concentrations and the transformed free plasma concentrations, using the R_b2p_ and f_ub in vivo_

Compound	Exposure scenario(s)	Reference	Plasma serum, or blood concentrations reported	Nominal internal concentrations (µM)^abc^			R_b2p_	F_ub in vivo_	Free internal plasma concentrations (µM)		
				Lowest	Mean	Highest			Lowest	Mean	Highest
E2	Female levels	Mayo Clinic Staff (2022a)	Plasma		5.51E–05	1.28E–03		0.08		4.18E–06	9.76E–05
E2	Male levels	Mayo Clinic Staff (2022a)	Plasma		3.67E–05	1.47E–04				2.79E–06	1.12E–05
T	Female levels	Mayo Clinic Staff (2022b)	Plasma		2.77E–06	2.08E–05		0.16		4.44E–07	3.33E–06
T	Male levels	Mayo Clinic Staff (2022b)	Plasma		8.32E–05	3.29E–04				1.33E–05	5.27E–05
BBzP	BBzP exposure biomonitoring 2–3 weeks after delivery in 36 Swedish women	(Högberg et al. 2008)	Blood	1.99E–04	1.16E–03	5.58E–03	0.80	0.04	8.05E–06	4.67E–05	2.26E–04
DBP	DBP exposure biomonitoring 2–3 weeks after delivery in 36 Swedish women	(Högberg et al. 2008)	Blood	9.34E–05	5.34E–03	4.05E–02	0.81	0.06	5.49E–06	3.13E–04	2.38E–03
EP	EP exposure biomonitoring in 60 healthy Danish young men	(Frederiksen et al. 2011)	Serum	2.65E–03		1.25E–01		0.20	5.19E–04		2.45E–02
EP	EP exposure biomonitoring in 58 fertile male patients of the Centre of Assisted Reproduction Pronatal, Prague	(Kolatorova Sosvorova et al. 2017)	Plasma		2.17E–03					4.24E–04	
EP	EP exposure biomonitoring in 150 healthy Malaysians	(Wiraagni et al. 2020)	Plasma		2.41E–03	5.72E–03				4.71E–04	1.12E–03
KEP	Affected workers Life Science Product Company, KEP production plant, Hopewell, USA (a)	(Cannon et al. 1978)	Blood	1.25E-02	3.50	1.63E+01	1.47	0.08	1.03E–03	2.89E–01	1.35
KEP	Unaffected workers Life Science Product Company, KEP production plant, Hopewell, USA (b)	(Cannon et al. 1978)	Blood	4.15E-03	8.31E-01	5.68			3.43E–04	6.85E–02	4.68E–01
KEP	Family members workers Life Science Product Company (c)	(Cannon et al. 1978)	Blood	4.15E-03	1.38E-01	5.40E-01			3.43E–04	1.14E–02	4.45E–02
KEP	Workers from another KEP production plant (Allied Chemical Corporation) (d)	(Cannon et al. 1978)	Blood	3.18E-03	8.31E-02	6.23E-01			2.63E–04	6.85E–03	5.14E–02
KEP	Neighborhood workers (e)	(Cannon et al. 1978)	Blood	4.15E-03	1.52E-02	4.29E-02			3.43E–04	1.26E–03	3.54E–03
KEP	Workers of a sewage treatment plant receiving effluents from the plant (f)	(Cannon et al. 1978	Blood	5.54E-03	1.11E-02	1.94E-02			4.57E–04	9.14E–04	1.60E–03
KEP	Cab driver (g)	(Cannon et al. 1978)	Blood		4.15E-03					3.43E–04	
KEP	Truck driver (h)	(Cannon et al. 1978)	Blood		6.00E-03					4.57E–04	
KEP	Hopewell residents (i)	(Cannon et al. 1978)	Blood	6.92E–03	1.52E–02	4.50E–02			5.71E–04	1.26E–03	3.71E–03
KEP	Occupational exposure, chemical plant workers, last day exposure	(Adir et al. 1978)	Serum	2.45E–01		4.30			2.02E–02		3.55E–01
KEP	Occupational exposure, chemical plant workers, after 6–7 m	(Adir et al. 1978)	Serum	7.54E–02		9.91E-01			6.22E–03		8.17E–02
KEP	General population within one mile radius chemical plant	(Adir et al. 1978)	Serum	1.02E–02		1.02E-01			8.41E–04		8.41E–03
KEP	Occupational exposure banana agriculture, Guadeloupe	(Multigner et al. 2006, 2008, 2016)	Blood	4.57E–03	1.48E–02	1.45E–01			3.77E–04	1.22E–03	1.19E–02
KEP	Occupational exposure nonagricultural sectors, Guadeloupe	(Multigner et al. 2006, 2008, 2016)	Blood	4.29E–03	1.16E–02	6.45E–02			3.54E–04	9.59E–04	5.32E–03
KEP	Estimated intake unpolluted area 1.9 (0.1–20.6) µg/day	(Guldner et al. 2010)	Blood		1.14E–03					9.37E–05	
KEP	Estimated intake polluted area 6.6 (1.0–22.2) µg/day	(Guldner et al. 2010)	Blood		1.41E–03					1.16E–04	
KEP	Consumption contaminated foodstuffs	(Kadhel et al. 2014)	Plasma	3.67E–04	7.95E–04	4.02E–02			3.03E–05	6.56E–05	3.31E–03
KEP	Consumption contaminated foodstuffs	Emeville et al. 2015	Plasma	3.47E–04	8.56E–04	9.42E–02			2.86E–05	7.06E–05	7.77E–03
o,p’-DDT	Occupational exposure to 26 workers spraying DDT Brazil	(Minelli and Ribeiro 1996)	Serum	1.43E–03		9.58E–03		0.03	3.63E–05		2.44E–04
o,p’-DDT	Environmental exposure to 193 children from polluted area Brazil	(Freire et al. 2013)	Serum	7.54E–04	2.38E–03	4.48E–03			1.92E–05	6.06E–05	1.14E–04
o,p’-DDT	Environmental exposure to 575 mothers from prospective birth cohort in the US	(Kezios et al. 2013)	Serum	2.04E–05	8.76E–04	9.09E–03			5.18E–07	2.23E–05	2.31E–04
BPA	Tolerable daily intake (TDI)	(Dent et al. 2019; Wetmore et al. 2015)	Plasma	1.55E–03	3.43E–03	6.91E–03		0.09	1.37E–04	3.02E–04	6.09E–04
COU	Biomonitoring of 246 healthy Chinese originating from two rural villages and two urban neighborhoods	(Liu et al. 2018)	Plasma	8.58E-03	1.01E-02	1.12E-02		0.08	6.87E–04	8.06E–04	8.96E–04
API	Single oral dose 17.77 ± 4.19 mg in 11 Healthy subjects	(Meyer et al. 2006)	Plasma	4.61E-02	1.27E-01	1.92E01		0.06	2.80E–03	7.69E–03	1.17E–02
API	Regular diet of 41 men from a cross-sectional study	(Bolarinwa and Linseisen 2005)	Plasma	7.40E–03	9.30E-03	5.25E-02			4.49E–04	5.65E–04	3.19E–03
ZEA	Calculated dietary intake 0.039 and 0.076 µg/kg/day in 260 healthy rural residents in China	(Fan et al. 2019)	Plasma	1.98E–04	4.93E–04	1.31E–03		0.07	1.39E–05	3.34E–05	8.90E–05
EE	Single oral dose 0.03 mg EE + 0.15 mg desogestrel to 24 healthy Indian females	(Nair et al. 2018)	Plasma	3.07E–04	3.12E–04	3.38E–04		0.05	1.66E–05	1.69E–05	1.82E–05
EE	Single oral dose 0.06 mg EE + 4 mg chlormadinone acetate to 20 healthy Caucasian females	(Bonn et al. 2009)	Plasma	2.80E–04	4.22E–04	5.63E–04			1.51E–05	2.28E–05	3.04E–05
DES	Single oral dose 2 mg to 12 healthy Chinese males	(Zhang et al. 2014)	Plasma	7.08E-03	1.12E-02	1.53E-02		0.04	6.54E–04	8.12E–04	9.71E–04
TAM	Single oral dose of 20 mg TAM to female early-stage breast cancer patients, in poor metabolizers	(Madlensky et al. 2011)	Serum	2.13E-01	3.83E-01	5.53E-01		0.04	8.97E–03	1.61E–02	2.33E–02
TAM	Single oral dose of 20 mg TAM female early-stage breast cancer patients, in intermediate metabolizers	(Madlensky et al. 2011)	Serum	1.94E-01	3.85E-01	5.75E-01			8.16E–03	1.62E–02	2.42E–02
TAM	Single oral dose of 20 mg TAM female early-stage breast cancer patients, in ultrarapid metabolizers	(Madlensky et al. 2011)	Serum	2.29E-01	3.86E-01	5.43E-01			9.62E–03	1.62E–02	2.28E–02

^a^Serum concentration were assumed to be equal to plasma concentrations

^b^Nominal internal blood concentrations were transformed to plasma concentration using the ADMET predicted blood to plasma ratio (R_b2p_) of the respective compound

^c^The units of the reported concentrations were transformed to the concentrations in µM using the molecular weight of the respective compound

**Table 4:** Evaluation of the exposure scenarios to the model compounds to be positive, negative or unknown for in vivo estrogenicity

Compound	Exposure scenario(s)	Reference	Corresponding in vivo estrogenic effects at exposure scenario reported	Safe reference level compound	In vivo estrogenicity at exposure scenario expected	Reasoning
E2	Female levels	Mayo Clinic Staff (2022a)	Endogenous levels estradiol		Yes	Endogenous estrogen
E2	Male levels	Mayo Clinic Staff (2022a)	Endogenous levels estradiol		Yes	Endogenous estrogen
T	Female levels	Mayo Clinic Staff (2022b)	Endogenous levels testosterone		No	Endogenous androgen
T	Male levels	Mayo Clinic Staff (2022b)	Endogenous levels testosterone		No	Endogenous androgen
BBzP	BBzP exposure biomonitoring 2–3 weeks after delivery in 36 Swedish women	(Högberg et al. 2008)	No information reported	TDI = 0.5 mg/kg^1^	Unknown	No intake levels of the exposure scenario were quantified, disabling comparison to the safe reference dose
DBP	DBP exposure biomonitoring 2–3 weeks after delivery in 36 Swedish women	(Högberg et al. 2008)	No information reported	TDI = 0.01 mg/kg^1^	Unknown	No intake levels of the exposure scenario were quantified, disabling comparison to the safe reference dose
EP	EP exposure biomonitoring in 60 healthy Danish young men	(Frederiksen et al. 2011)	No information reported	Group ADI with methyl paraben and their sodium salts = 0—10 mg/kg bw^2^	Unknown	No intake levels of the exposure scenario were quantified, disabling comparison to the safe reference dose
EP	EP exposure biomonitoring in 58 fertile male patients of the Centre of Assisted Reproduction Pronatal, Prague	(Kolatorova Sosvorova et al. 2017)	No information reported		Unknown	No intake levels of the exposure scenario were quantified, disabling comparison to the safe reference dose
EP	EP exposure biomonitoring in 150 healthy Malaysians	(Wiraagni et al. 2020)	No information reported		Unknown	No intake levels of the exposure scenario were quantified, disabling comparison to the safe reference dose
KEP	Affected workers Life Science Product Company, KEP production plant, Hopewell, USA (a)	(Cannon et al. 1978)	Aside from some reports on toxic effects in the testis, no conclusion on onset of estrogenic effects in the subjects was given	ADI = 0.5 µg/kg bw^3^ ARfD = 10 µg/kg bw^3^ NOEL in men based on a clinically relevant decrease in sperm count = 0.1–0.5 mg/L = 0.2–1.0 µM in blood^4^ Using the R_b2p_ of 1.47 the NOEL in plasma (assumed to be equal as in serum) = 0.14 – 0.68 µM	Yes	No intake levels of the exposure scenario were quantified, disabling comparison to the ADI or ARfD. Assuming a male population of the subjects, comparison to the NOEL in blood indicates that in vivo estrogenic effects may be expected
KEP	Unaffected workers Life Science Product Company, KEP production plant, Hopewell, USA (b)	(Cannon et al. 1978)	No information reported		Yes	No intake levels of the exposure scenario were quantified, disabling comparison to the ADI or ARfD. Assuming a male population of the subjects, comparison to the NOEL in blood indicates that in vivo estrogenic effects may be expected
KEP	Family members workers Life Science Product Company (c)	(Cannon et al. 1978)	No information reported		Yes	No intake levels of the exposure scenario were quantified, disabling comparison to the ADI or ARfD. Assuming a male population of the subjects, comparison to the NOEL in blood indicates that in vivo estrogenic effects may be expected
KEP	Workers from another KEP production plant (Allied Chemical Corporation) (d)	(Cannon et al. 1978)	No information reported		Yes	No intake levels of the exposure scenario were quantified, disabling comparison to the ADI or ARfD. Assuming a male population of the subjects, comparison to the NOEL in blood indicates that in vivo estrogenic effects may be expected
KEP	Neighborhood workers (e)	(Cannon et al. 1978)	No information reported		No	No intake levels of the exposure scenario were quantified, disabling comparison to the ADI or ARfD. Assuming a male population of the subjects, comparison to the NOEL in blood indicates that no in vivo estrogenic effects are expected
KEP	Workers of a sewage treatment plant receiving effluents from the plant (f)	(Cannon et al. 1978)	No information reported		No	No intake levels of the exposure scenario were quantified, disabling comparison to the ADI or ARfD. Assuming a male population of the subjects, comparison to the NOEL in blood indicates that no in vivo estrogenic effects are expected
KEP	Cab driver (g)	(Cannon et al. 1978)	No information reported		No	No intake levels of the exposure scenario were quantified, disabling comparison to the ADI or ARfD. Assuming a male population of the subjects, comparison to the NOEL in blood indicates that no in vivo estrogenic effects are expected
KEP	Truck driver (h)	(Cannon et al. 1978)	No information reported		No	No intake levels of the exposure scenario were quantified, disabling comparison to the ADI or ARfD. Assuming a male population of the subjects, comparison to the NOEL in blood indicates that no in vivo estrogenic effects are expected
KEP	Hopewell residents (i)	(Cannon et al. 1978)	No information reported		No	No intake levels of the exposure scenario were quantified, disabling comparison to the ADI or ARfD. Assuming a male population of the subjects, comparison to the NOEL in blood indicates that no in vivo estrogenic effects are expected
KEP	Occupational exposure, chemical plant workers, last day exposure	(Adir et al. 1978)	No information reported		Yes	No intake levels of the exposure scenario were quantified, disabling comparison to the ADI or ARfD. Assuming a male population of the subjects, comparison to the transformed NOEL in serum indicates that in vivo estrogenic effects may be expected
KEP	Occupational exposure, chemical plant workers, after 6–7 m	(Adir et al. 1978)	No information reported		Yes	No intake levels of the exposure scenario were quantified, disabling comparison to the ADI or ARfD. Assuming a male population of the subjects, comparison to the transformed NOEL in serum indicates that in vivo estrogenic effects may be expected
KEP	General population within one mile radius chemical plant	(Adir et al. 1978)	No information reported		No	No intake levels of the exposure scenario were quantified, disabling comparison to the ADI or ARfD. Assuming a male population of the subjects, comparison to the transformed NOEL in serum indicates that no in vivo estrogenic effects are expected
KEP	Occupational exposure banana agriculture, Guadeloupe	(Multigner et al. 2006, 2008, 2016)	No significant difference in sperm and hormone characteristics were found		No	It was reported that no corresponding in vivo estrogenic effects at exposure scenario occurred
KEP	Occupational exposure nonagricultural sectors, Guadeloupe	Multigner et al. 2006, 2008, 2016	No significant difference in sperm and hormone characteristics were found		No	It was reported that no corresponding in vivo estrogenic effects at exposure scenario occurred
KEP	Estimated intake unpolluted area 1.9 (0.1—20.6) µg/day	(Guldner et al. 2010)	No information reported		No	Estimated intake levels were below the ADI and ARfD
KEP	Estimated intake polluted area 6.6 (1.0 -22.2) µg/day	(Guldner et al. 2010)	No information reported		No	Estimated intake levels were below the ADI and ARfD
KEP	Consumption contaminated foodstuffs	(Kadhel et al. 2014)	Maternal plasma levels of > 0.52 ng/mL KEP were related to changes in the length of gestation and risk of preterm birth		Yes	At the highest reported maternal plasma concentrations it was reported that corresponding in vivo estrogenic effects occurred
KEP	Consumption contaminated foodstuffs	(Emeville et al. 2015)	No information reported		No	Exposure in the control subjects in a case–control study on the relationship of prostate cancer
o,p’-DDT	Occupational exposure to 26 workers spraying DDT Brazil	(Minelli and Ribeiro 1996)	No information reported	ADI = 0.01 mg/kg^5^	Unknown	No intake levels of the exposure scenario were quantified, disabling comparison to the safe reference dose
o,p’-DDT	Environmental exposure to 193 children from polluted area Brazil	(Freire et al. 2013)	No information reported		Unknown	No intake levels of the exposure scenario were quantified, disabling comparison to the safe reference dose
o,p’-DDT	Environmental exposure to 575 mothers from prospective birth cohort in the US	(Kezios et al. 2013)	No information reported		Unknown	No intake levels of the exposure scenario were quantified, disabling comparison to the safe reference dose
BPA	Tolerable daily intake (TDI)	(Dent et al. 2019; Wetmore et al. 2015)	No information reported	TDI = 4 µg/kg bw/d^6^	No	Exposure scenario at the TDI so no in vivo estrogenic effects expected
COU	Biomonitoring of 246 healthy Chinese originating from two rural villages and two urban neighborhoods	(Liu et al. 2018)	No information reported		Unknown	No safe reference dose is available and no intake levels of the exposure scenario were quantified
API	Single oral dose 17.77 ± 4.19 mg in 11 Healthy subjects	(Meyer et al. 2006)	No information reported		Unknown	No safe reference dose is available to compare reported intake levels
API	Regular diet of 41 men from a cross-sectional study	(Bolarinwa and Linseisen 2005)	No information reported		Unknown	No safe reference dose is available and no intake levels reported
ZEA	Calculated dietary intake 0.039 and 0.076 µg/kg/day in 260 healthy rural residents in China	(Fan et al. 2019)	No information reported	TDI = 0.25 µg/kg bw/d^7^	No	Calculated dietary intake was below the TDI
EE	Single oral dose 0.03 mg EE + 0.15 mg desogestrel to 24 healthy Indian females	(Nair et al. 2018)	Therapeutic dose		Yes	Exposure scenario embodies the therapeutic use of EE
EE	Single oral dose 0.06 mg EE + 4 mg chlormadinone acetate to 20 healthy Caucasian females	(Bonn et al. 2009)	Therapeutic dose		Yes	Exposure scenario embodies the therapeutic use of EE
DES	Single oral dose 2 mg to 12 healthy Chinese males	(Zhang et al. 2014)	Therapeutic dose		Yes	Exposure scenario embodies the therapeutic use of DES
TAM	Single oral dose of 20 mg TAM to female early-stage breast cancer patients, in poor metabolizers	(Madlensky et al. 2011)	Therapeutic dose		Yes	Exposure scenario embodies the therapeutic use of TAM
TAM	Single oral dose of 20 mg TAM female early-stage breast cancer patients, in intermediate metabolizers	(Madlensky et al. 2011)	Therapeutic dose		Yes	Exposure scenario embodies the therapeutic use of TAM
TAM	Single oral dose of 20 mg TAM female early-stage breast cancer patients, in ultrarapid metabolizers	(Madlensky et al. 2011)	Therapeutic dose		Yes	Exposure scenario embodies the therapeutic use of TAM

^a^Silano et al. (2019)

^b^Anton et al. (2004)

^c^French Agency for Food, Environmental and Occupational Health and Safety (ANSES) (2018a, b), (2019)

^d^Guzelian (1992)

^e^Joint Food Agricultural Organization/World Health Organization Meeting on Pesticide Residues (JMPR) (2000)

^f^Bolognesi rt al. (2015)

^g^Alexander et al. (2011)

TDI = Tolerable daily intake. ADI = Acceptable daily intake. ARfD = Acute reference dose. NOEL = No observed effect level

Step 4: Evaluation of the selected comparator compound genistein based on available negative exposure scenarios and comparison of corresponding internal concentrations to derived BMCL_05_ (no effect level) values from the in vitro assays.

**Fig. 3:**
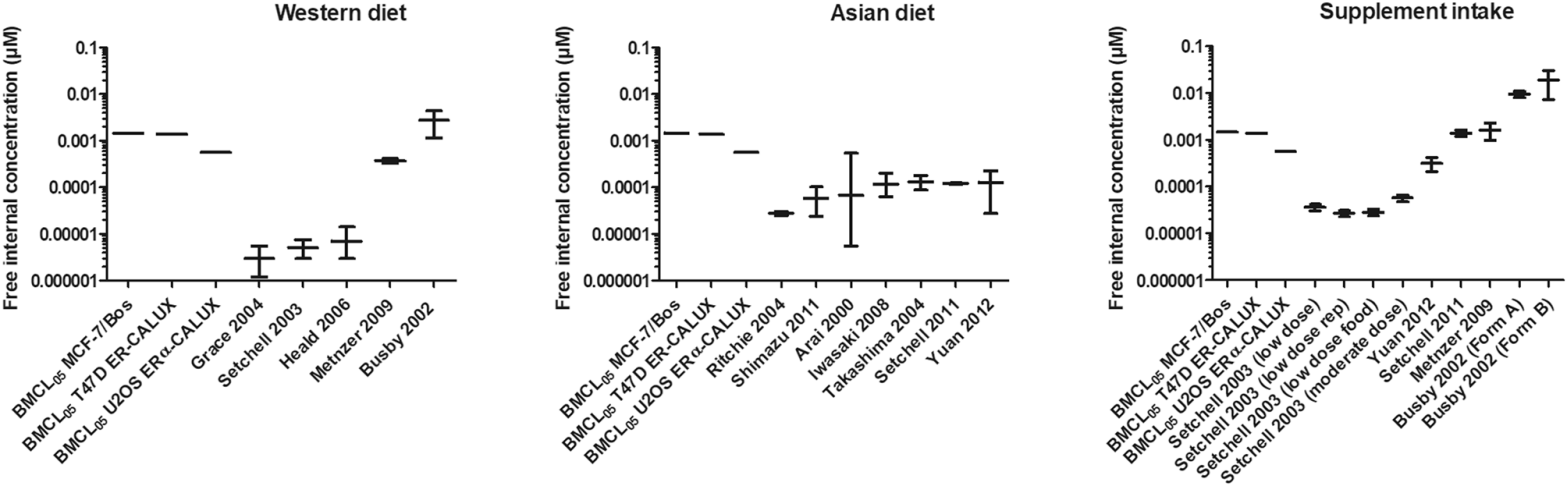
Comparison of the free in vivo BMCL_05_ values based on the MCF-7/Bos proliferation assay, T47D ER-CALUX assay, or U2OS ERα-CALUX assay (first 3 bars in each graph) and literature reported free in vivo internal concentrations of GEN, including the variability, following a Western diet, an Asian diet, or supplement intake in humans as derived from the respective references

GEN was selected as comparator compound based on the large amount of available data on exposures that result in negative outcomes for in vivo estrogenicity in humans, such as the exposures resulting from dietary intake levels which are indicated to be conservative and health protective in humans, and correspond to a Western diet, an Asian diet, or GEN supplements (Becker et al. 2015) (Supplementary material S1). The results of the benchmark dose modelling to derive the BMCL_05_ values are presented in Supplementary material S2 and the derived nominal and transformed free in vitro BMCL_05_ values of GEN (considered equal to safe free in vivo BMCL_05_ values) are compiled in Table 5 . The free in vivo BMCL_05_ values were compared to the free human internal concentrations of GEN transformed from the literature reported nominal concentrations at the reported exposures using the f_ub in vivo_ (Fig. 3).

**Table 5:** Nominal and transformed free EC_50_ and BMCL_05_ values, using the f_ub in vitro*,*_ of comparator compound GEN based on the in vitro MCF-7/Bos proliferation assay, T47D ER-CALUX assay, or U2OS ERα-CALUX assay and the corresponding EAR_comparator_ values calculated using Eq. 1

Assay	Nominal EC_50_ (µM)	Nominal in vitro BMCL_05_ (µM)	F_ub in vitro_ (comparator)	Free EC_50_ (µM)	Free in vitro BMCL_05_ = Free in vivo BMCL_05_ (µM)	EAR_comparator_
MCF-7/BOS proliferation	4.60E–02	3.48E–03	0.42	1.93E-02	1.46E–03	7.59E–02
T47D ER-CALUX	1.30E-01	3.29E–03		5.46E-02	1.38E–03	2.53E–02
U2OS ERα-CALUX	6.80E–02	1.34E–03		2.86E-02	5.60E–04	1.97E–02

The free internal concentrations resulting from a western diet ranged from 3.36 × 10^–6^ ± 2.00 × 10^–6^ µM (Grace et al. 2004) to 2.76 × 10^–3^ ± 1.60 × 10^–3^ µM (Busby et al. 2002), indicating orders of magnitude variation, although all concentrations were substantially lower than the free BMCL_05_ values derived from the in vitro assays. GEN intake reported from an Asian diet resulted in free internal concentrations ranging from 2.76 × 10^–5^ ± 0.30 × 10^–5^ µM (Ritchie et al. 2004) to 1.26 × 10^–4^ ± 0.99 × 10^–4^ µM (Yuan et al. 2012), showing less variance, with still all values being below the free BMCL_05_ values derived from the in vitro assays (Fig. 3). Supplement intake resulted in reported free internal GEN concentrations ranging from 2.68 × 10^–5^ ± 0.39 × 10^–5^ µM (Setchell et al. 2003) to 1.89 × 10^–2^ ± 1.16 × 10^–2^ µM (Busby et al. 2002), showing variance due to the different intake levels of GEN when using different supplements at different dosing regimens. The highest internal concentration was reported from supplement intake of GEN by Busby et al. (2002) and was 13- to 34-fold higher than the free in vivo BMCL_05_ values of GEN. However, because the study also reported that there were no estrogenic effects observed in the 30 male volunteers studied it can be concluded that these results further support that also the exposure to the comparator GEN that results in an internal free concentration equal to the in vitro free BMCL_05_ can be considered safe and is adequate to calculate the EAR_comparator_ in the DCR approach.

Step 5: Derivation of EC_50_ values (as effect levels) from the in vitro concentration-response curves and conversion to free concentrations.

The free EC_50_ values as effect level of the compounds were derived from the concentration-response curves obtained in the MCF-7/Bos proliferation assay, T47D ER-CALUX assay, and U2OS ERα-CALUX assay (Wang et al. 2014), transforming the nominal concentrations to the free concentrations using the f_ub in vitro_ (Table 6). Note that testosterone had no response in the T47D ER-CALUX assay.

**Table 6:** EC_50_ values of the test compounds derived from the MCF-7/Bos proliferation assay, T47D ER-CALUX assay, and U2OS ERα-CALUX assay. The nominal EC_50_ values as taken from Wang et al. (2014) were transformed to the free EC_50_ values using the f_ub in vitro_

Compound	Nominal EC_50_ (µM)			F_ub in vitro_ (test)	Free EC_50_ (µM)		
	MCF-7/BOS proliferation	T47D ER-CALUX	U2OS ERα-CALUX		MCF-7/BOS proliferation	T47D ER-CALUX	U2OS ERα-CALUX
E2	2.00E–05	5.00E–06	8.60E–06	0.42	8.45E–06	2.11E–06	3.63E–06
T	2.10		8.50E–01	0.48	1.00		4.04E−01
BBzP	2.00	5.70	1.00E+01	0.40	8.00E−01	2.28	4.00
DBP	3.00	1.70E+01	1.90E+01	0.41	1.24	7.00	7.82
EP	1.40E+01	5.50	4.20E+01	0.50	6.96	2.74	2.0.9E0+1
KEP	4.90E+01	6.70E–01	8.50E−01	0.43	2.09E−01	2.86E−01	3.63E−01
o,p’-DDT	3.80E−01	4.10E–01	7.20E−01	0.39	1.49E−01	1.60E−01	2.80E−01
BPA	3.60E−01	7.70E–01	2.20E−01	0.43	1.55E−01	3.31E−01	9.46E−02
COU	1.30E–02	5.20E–03	4.40E–02	0.43	5.53E−03	2.21E–03	1.87E−02
API	6.20E−01	4.10E–01	5.80E–01	0.41	2.56E−01	1.69E–01	2.40E−01
ZEA	1.50E–04	2.30E–04	4.20E–04	0.42	6.26E−05	9.60E–05	1.75E–04
EE	9.70E–06	2.70E–06	5.60E–06	0.41	3.96E–06	1.10E–06	2.29E–06
DES	3.80E–05	1.80E–05	8.10E–05	0.40	1.52E–05	7.18E–06	3.23E–05
TAM	4.10E–03	1.50E–02	2.10E–02	0.40	1.65E–03	6.02E–03	8.43E–03

Step 6: Calculation of the EAR_comparator_ values.

With free BMCL_05_ and free EC_50_ values of GEN derived from data from the MCF-7/Bos proliferation assay, T47D ER-CALUX assay, and U2OS ERα-CALUX assay (Step 5), the EAR_comparator_ values were calculated using Eq. 1 and are listed in Table 5. The EAR_comparator_ values derived from the 3 assays increased in the order U2OS ERα-CALUX assay < T47D ER-CALUX assay < MCF-7/Bos proliferation assay.

Step 7: Calculation of EAR_test_ values.

Using the free internal concentrations at the respective exposure scenario of the model compounds and their free EC_50_ values (Table 6) derived from the data from the 3 in vitro estrogenicity assays, the EAR_test_ values were calculated following Eq. 2 and are compiled in Table 7. When information on the variability of the exposure was available, the corresponding lowest, mean, and highest EAR_test_ value was calculated.

**Table 7:** EAR_test_ values of the test compounds based on the MCF-7/Bos proliferation assay, T47D ER-CALUX assay, and U2OS ERα-CALUX assay calculated using Eq. 2

Compound	Exposure scenario(s)	Reference	EAR_test_ MCF-7/BOS proliferation			EAR_test_ T47D ER-CALUX			EAR_test_ U2OS ERα-CALUX		
			Lowest	Mean	Highest	Lowest	Mean	Highest	Lowest	Mean	Highest
E2	Female levels	Mayo Clinic staff (2022a)		4.95E–01	1.16E + 01		1.98	4.62E + 01		1.15	2.69E + 01
E2	Male levels	Mayo Clinic staff (2022a)		3.30E–01	1.32		1.32	5.28		7.68E–01	3.07
T	Female levels	Mayo Clinic staff (2022b)		4.45E–07	3.34E–06					1.10E–06	8.25E–06
T	Male levels	Mayo Clinic staff (2022b)		1.34E–05	5.29E–05					3.30E–05	1.31E–04
BBzP	BBzP exposure biomonitoring 2–3 weeks after delivery	(Högberg et al. 2008)	1.01E–05	5.84E–05	2.82E–04	3.53E–06	2.05E–05	9.89E–05	2.01E–06	1.17E–05	5.63E–05
DBP	DBP exposure biomonitoring 2–3 weeks after delivery	(Högberg et al. 2008)	4.44E–06	2.54E–04	1.92E–03	7.84E–07	4.48E–05	3.40E–04	7.01E–07	4.01E–05	3.04E–04
EP	EP exposure biomonitoring	(Frederiksen et al. 2011)	7.45E–05		3.52E–03	1.90E–04		8.96E–03	2.48E–05		1.17E–03
EP	EP exposure biomonitoring	(Kolatorova Sosvorova et al. 2017)		6.09E–05			1.55E–04			2.03E–05	
EP	Daily use of products and from the environment	(Wiraagni et al. 2020)		6.77E–05	1.61E–04		1.72E–04	4.09E–04		2.26E–05	5.36E–05
KEP	Affected workers Life Science Product Company, KEP production plant, Hopewell, USA (a)	(Cannon et al. 1978)	4.92E–03	1.38	6.45	3.60E–03	1.01	4.72	2.84E–03	7.97E–01	3.72
KEP	Unaffected workers Life Science Product Company, KEP production plant, Hopewell, USA (b)	(Cannon et al. 1978)	1.64E–03	3.28E–01	2.24	1.20E–03	2.40E–01	1.64	9.45E–04	1.89E–01	1.29
KEP	Family members workers Life Science Product Company (c)	(Cannon et al. 1978)	1.64E–03	5.46E–02	2.13E–01	1.20E–03	4.00E–02	1.56E–01	9.45E–04	3.15E–02	1.23E–01
KEP	Workers from another KEP production plant (Allied Chemical Corporation) (d)	(Cannon et al. 1978)	1.26E–03	3.28E–02	2.46E–01	9.19E–04	2.40E–02	1.80E–01	7.25E–04	1.89E–02	1.42E–01
KEP	Neighborhood workers (e)	(Cannon et al. 1978)	1.64E–03	6.01E–03	1.69E–02	1.20E–03	4.40E–03	1.24E–02	9.45E–04	3.47E–03	9.77E–03
KEP	Workers of a sewage treatment plant receiving effluents from the plant (f)	(Cannon et al. 1978)	2.19E–03	4.37E–03	7.65E–03	1.60E–03	3.20E–03	5.59E–03	1.26E–03	2.52E–03	4.41E–03
KEP	Cab driver (g)	(Cannon et al. 1978)		1.64E–03			1.20E–03			9.45E–04	
KEP	Truck driver (h)	(Cannon et al. 1978)		2.19E–03			1.60E–03			1.26E–03	
KEP	Hopewell residents (i)	(Cannon et al. 1978)	2.73E–03	6.01E–03	1.78E–02	2.00E–03	4.40E–03	1.30E–02	1.58E–03	3.47E–03	1.02E–02
KEP	Occupational exposure, chemical plant workers, last day exposure	(Adir et al. 1978)	9.65E–02		1.70	7.06E–02		1.24	5.56E–02		9.78E–01
KEP	Occupational exposure, chemical plant workers, after 6–7 m	(Adir et al. 1978)	2.98E–02		3.91E–01	2.18E–02		2.86E–01	1.72E–02		2.25E–01
KEP	General population within one mile radius chemical plant	(Adir et al. 1978)	4.02E–03		4.02E–02	2.94E–03		2.94E–02	2.32E–03		2.32E–02
KEP	Occupational exposure banana agriculture, Guadeloupe	(Multigner et al. 2006, 2008, 2016)	1.80E–03	5.85E–03	5.71E–02	1.32E–03	4.28E–03	4.18E–02	1.04E–03	3.37E–03	3.29E–02
KEP	Occupational exposure nonagricultural sectors, Guadeloupe	Multigner et al. 2006, 2008, 2016	1.69E–03	4.59E–03	2.55E–02	1.24E–03	3.36E–03	1.86E–02	9.77E–04	2.65E–03	1.47E–02
KEP	Estimated intake unpolluted area 1.9 (0.1—20.6) µg/day	(Guldner et al. 2010)		4.48E–04			3.28E–04			2.58E–04	
KEP	Estimated intake polluted area 6.6 (1.0 -22.2) µg/day	(Guldner et al. 2010)		5.57E–04			4.08E–04			3.21E–04	
KEP	Consumption contaminated foodstuffs	(Kadhel et al. 2014)	1.45E–04	3.14E–04	1.58E–02	1.06E–04	2.29E–04	1.16E–02	8.35E–05	1.81E–04	9.13E–03
KEP	Consumption contaminated foodstuffs	(Emeville et al. 2015)	1.37E–04	3.38E–04	3.96E–02	1.00E–04	2.47E–04	2.89E–02	7.88E–05	1.95E–04	2.28E–02
o,p’-DDT	Occupational exposure spraying DDT Brazil	(Minelli and Ribeiro 1996)	3.38E–04		2.27E–03	3.13E–04		2.10E–03	1.78E–04		1.20E–03
o,p’-DDT	Environmental exposure from polluted area Brazil	(Freire et al. 2013)	1.79E–04	5.65E–04	1.06E–03	1.66E–04	5.23E–04	9.84E–04	9.43E–05	2.98E–04	5.61E–04
o,p’-DDT	Environmental exposure US	(Kezios et al. 2013)	4.83E–06	2.08E–04	2.15E–03	4.47E–06	1.92E–04	2.00E–03	2.55E–06	1.10E–04	1.14E–03
BPA	Tolerable daily intake (TDI)	(Dent et al. 2019; Wetmore et al. 2015)	8.82E–04	1.95E–03	3.93E–03	4.12E–04	9.13E–04	1.84E–03	1.44E–03	3.19E–03	6.43E–03
COU	Biomonitoring	(Liu et al. 2018)	1.24E–01	1.46E–01	1.62E–01	3.11E–01	3.65E–01	4.05E–01	3.67E–02	4.31E–02	4.79E–02
API	Single oral dose 17.77 ± 4.19 mg	(Meyer et al. 2006)	3.53E–02	3.00E–02	4.56E–02	3.53E–02	4.54E–02	6.89E–02	3.53E–02	3.21E–02	4.87E–02
API	Regular diet	(Bolarinwa and Linseisen 2005)	2.07E–02	2.21E–03	1.24E–02	2.07E–02	3.33E–03	1.88E–02	2.07E–02	2.36E–03	1.33E–02
ZEA	Calculated dietary intake 39 and 76 ng/kg/day, mean and max, respectively	(Fan et al. 2019)	1.97E–01	5.34E–01	1.42	1.29E–01	3.48E–01	9.27E–01	7.04E–02	1.91E–01	5.08E–01
EE	Single oral dose 0.03 mg EE + 0.15 mg desogestrel	(Nair et al. 2018)	4.18	4.25	4.60	1.50E + 01	1.53E + 01	1.65E + 01	7.23	7.37	7.97
EE	Single oral dose 0.06 mg EE + 4 mg chlormadinone acetate	(Bonn et al. 2009)	3.82	5.74	7.66	1.37E + 01	2.06E + 01	2.75E + 01	6.61	9.94	1.33E + 01
DES	Single oral dose 2 mg	(Zhang et al. 2014)	1.80E + 01	2.85E + 01	3.89E + 01	3.81E + 01	6.01E + 01	8.22E + 01	8.47	1.34E + 01	1.83E + 01
TAM	Single oral dose of 20 mg TAM, in poor metabolizers	(Madlensky et al. 2011)	5.45	9.79	1.41E + 01	1.49	2.68	3.86	1.06	1.91	2.76
TAM	Single oral dose of 20 mg TAM, in intermediate metabolizers	(Madlensky et al. 2011)	4.96	9.83	1.47E + 01	1.36	2.69	4.02	9.69E–01	1.92	2.87
TAM	Single oral dose of 20 mg TAM, in ultrarapid metabolizers	(Madlensky et al. 2011)	5.85	9.87	1.39E + 01	1.60	2.70	3.80	1.14	1.93	2.71

Step 8: Calculation of DCR values.

**Fig. 4:**
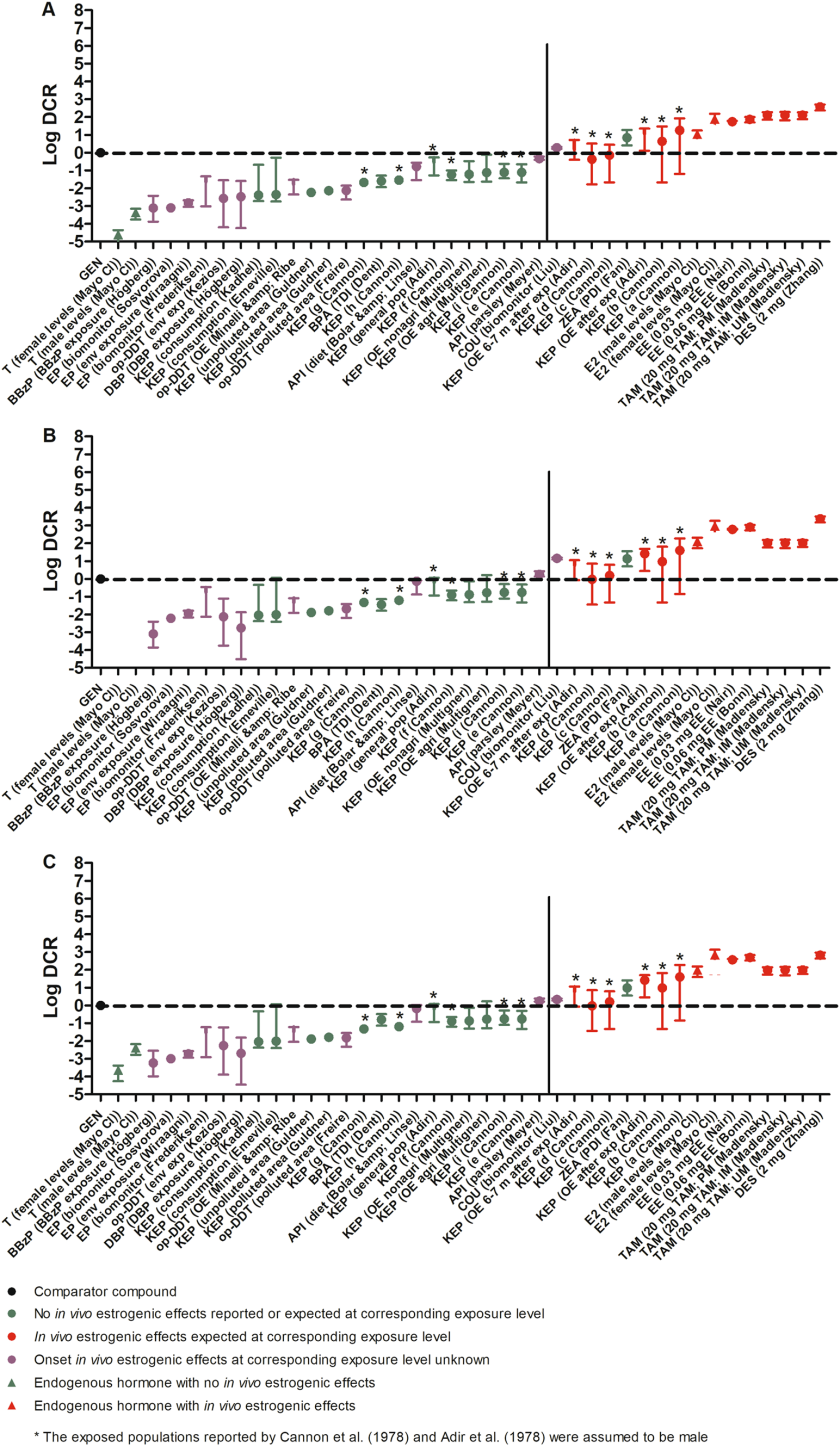
The DCRs of a series of exposures to 14 model compounds including endogenous hormones, phthalates, ethyl paraben, pesticides, bisphenol A, phytoestrogens, the mycotoxin zearalenone, and drugs with information regarding accompanying in vivo estrogenic effects calculated using EAR_comparator_ values of GEN (Table 5) based on **A**. the MCF-7/Bos proliferation assay, **B** the T47D ER-CALUX assay, and **C** the U2OS ERα-CALUX assay. The mean DCR values are presented as symbols and, when information on the variability was available, the lowest and highest DCR values as the lowest and highest whiskers, respectively. The DCRs of comparator GEN are represented as black circles and by definition equal to 1 (log DCR = 0). The DCRs of model compound exposure scenarios where no *in vivo* estrogenic effects are expected (see Table 4) are presented as green circles. The DCRs of test compound exposure scenarios for which in vivo estrogenic effects are expected (see Table 4) are presented as red circles. The DCRs of test compound scenarios for which the in vivo estrogenic effects are unknown (see Table 4) are presented as purple circles. The DCRs for the endogenous hormone levels of testosterone and estradiol are presented as green and red triangles, respectively. The dotted horizontal lines display the DCR of 1 (log DCR = 0) whereas the solid vertical lines separate the exposures with mean DCR values ≤ 1 from those with mean DCR values > 1. See Table 1 for compound abbreviations.

With the established EAR_test_ values for the multiple exposure scenarios for the selected model compounds (Table 7) and the in vitro-based EAR_comparator_ values of GEN (Table 5), the DCR values were calculated using Eq. 3 using data from the MCF-7/Bos proliferation assay (Fig. 4A), T47D ER-CALUX assay (Fig. 4B), and U2OS ERα-CALUX assay (Fig. 4C).

Comparison of the results presented in Fig. 4A–C reveals that the exposure scenarios with a DCR value ≤ 1 are the same when based on the 3 in vitro estrogenic activity assays and the corresponding in vitro-based EAR_comparator_ values of GEN, and this also holds true for the exposure scenarios with a DCR value > 1. The derived DCR values were relatively lower when based on the MCF-7/Bos proliferation assay (Fig. 4A). The EAR_comparator_ from this assay was highest compared to the other in vitro assays (Table 5) which indicates that the corresponding DCR values from the MCF-7/Bos proliferation assay appear least conservative so that on the basis of this assay it is more likely to conclude an exposure is safe.

Step 9: Evaluation of the DCR-based predictions of the selected exposure scenarios to the test compounds.

To evaluate the calculated DCR values, a comparison was made to actual knowledge on the corresponding in vivo estrogenic effects at the respective exposure levels (Table 4), also including endogenous hormone levels of androgen T and estrogen E2 in males and females. Indeed, the male and female levels of T (green triangles) and E2 (red triangles) had DCR values of respectively < 1 and > 1 indicating they are negative and positive for inducing in vivo estrogenicity. In adult males, E2 regulates efferent duct and prostate functioning and the flow of sperm from testis to the epididymis, thus playing a role in male fertility and reproductive functioning (Hess and Cooke 2018). All exposure scenarios which were expected based on existing knowledge to be positive for in vivo estrogenic effects (red circles) had a DCR > 1. There was one false positive value that related to the evaluated exposure scenario for ZEA (Fan et al. 2019) wherefrom no in vivo estrogenic effects are expected but still resulted in a DCR > 1. All exposures which were negative for in vivo estrogenicity (green circles) had a DCR ≤ 1.

Step 9a: Evaluation of exposures to ZEA.

**Fig. 5:**
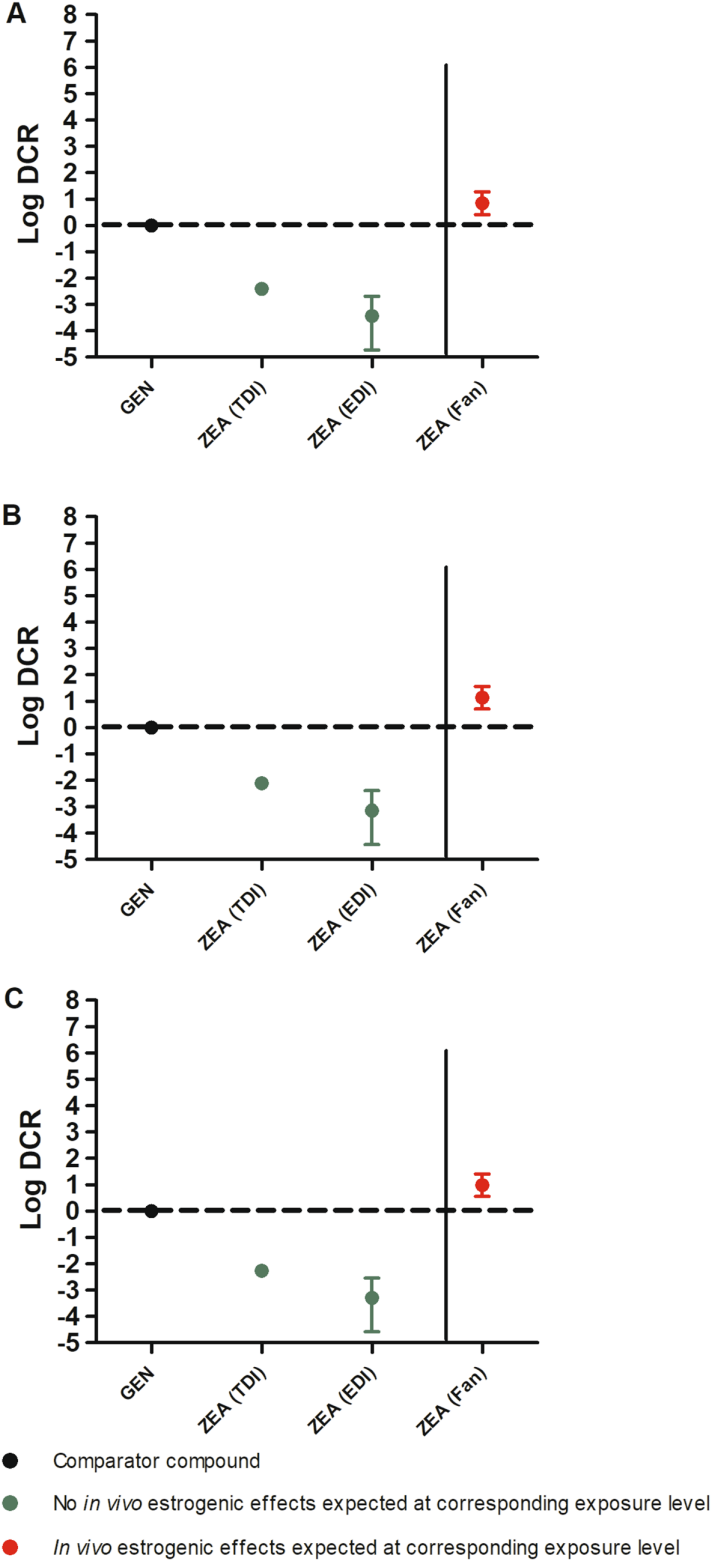
The DCRs of the TDI, EDI and the reported exposure scenario (Fan et al. 2019) of ZEA calculated using EAR_comparator_ values of GEN (Table 5) based on **A** the MCF-7/Bos proliferation assay, **B** the T47D ER-CALUX assay, and **C** the U2OS ERα-CALUX assay. The mean DCR values are presented as circles and, when information on the variability was available, the lowest and highest DCR values as the lowest and highest whiskers, respectively. The DCRs of comparator GEN are represented as black circles and by definition equal to 1 (log DCR = 0). The DCRs of the exposure scenarios to ZEA where no in vivo estrogenic effects are expected are presented as green circles. The DCRs of exposure scenarios to ZEA for which in vivo estrogenic effects are expected are presented as red circles. The dotted horizontal lines display the DCR of 1 (log DCR = 0) whereas the solid vertical lines separate the exposures with mean DCR values ≤ 1 from those with mean DCR values > 1

A further analysis of the false positive result for the exposure scenario of ZEA (Fan et al. 2019) was performed. The corresponding DCR values were > 1, which suggests that there would be a risk for in vivo estrogenicity at this exposure. At the reported internal exposures, Fan et al. (2019) calculated a probable daily intake (PDI) of 3.9 × 10^–2^– 7.6 × 10^–2^ µg/kg bw/day which is 3.2- to 6.4-fold lower than the tolerable daily intake (TDI) of ZEA of 0.25 µg/kg bw/day established based on the no observed effect level (NOEL) for estrogenic effects of ZEA and its metabolites on the ovary, uterus, and vulva in pigs (Alexander et al. 2011). Based on this result this exposure scenario was expected to not result in estrogenicity, indicating that the positive DCR based prediction or this scenario to be apparently false. However, this PDI reported by Fan et al. (2019) was calculated using simple kinetics and may not provide an adequate estimation of the corresponding external dose levels that correspond with the reported plasma concentrations of ZEA. Using physiologically based kinetic (PBK) modelling, we aimed to obtain a more accurate dose prediction of ZEA at the reported plasma concentrations of Fan et al. (2019). To this purpose, the PBK model describing ZEA kinetics in humans developed and validated by Mendez-Catala et al. (2021) (PBK model code available in the Supplementary data of Mendez-Catala et al. (2021)) was used to predict the external dose levels of ZEA at the internal exposure reported by Fan et al. (2019), using Berkeley Madonna 10.4.2 (UC Berkeley, CA, USA) with the Rosenbrock’s algorithm for stiff systems. The PBK model of ZEA includes the metabolic transformation and kinetics of the more estrogenic active metabolite α-zearalenol (α-ZEL). The nominal plasma concentrations of ZEA (1.98 × 10^–4^—0.13 × 10^–4^ µM) were transformed using the ADMET predicted R_b2p_ of ZEA (0.89) to the corresponding nominal blood concentrations (1.76 × 10^–4^—0.12 × 10^–4^ µM). Next, the corresponding doses of ZEA that would be required to reach these nominal blood concentrations were predicted using the PBK model. The predicted doses amounted to 335 – 2200 µg/kg bw/day and appear 3 to 4 orders of magnitude higher than the calculated PDI of Fan et al. (2019). These dose levels are also higher than the TDI of ZEA indicating that this exposure to ZEA can be expected to result in estrogenicity. This indicates the DCR values being > 1 would be in line with what would be expected indicating the data point for ZEA to be a real positive. It is of interest to note that Mendez-Catala et al. (2021) used the PBK model to predict the free plasma concentrations of ZEA at its TDI and at the estimated daily intake (EDI) ranging from 2.40 × 10^–3^ to 29 × 10^–2^ µg/kg bw/day (Alexander et al. 2011). The predicted free plasma concentration at the TDI amounted to 1.88 × 10^–8^ µM and at the EDI to 9.00 × 10^–9^— 9.00 × 10^–11^ µM (Mendez-Catala et al. 2021). Thus, the plasma concentrations reported by Fan et al. (2019) appear 6 orders of magnitude higher than these predicted plasma concentrations at the TDI also indicating that the exposure scenario reported by Fan et al. (2019) represents a scenario that would likely test positive for estrogenicity. To further illustrate thus by the DCR approach, these free plasma concentrations resulting from exposure at the EDI or TDI were used to calculate the corresponding EAR_test_ (Supplementary material S3) and DCR values using GEN as comparator compound. The DCRs at the EDI and TDI of ZEA were indeed < 1 (Fig. 5) and thus no in vivo estrogenicity is expected and confirms the safety of the EDI and TDI of ZEA. For comparison, the DCR for the exposure scenario of Fan et al. (2019) now colored red instead of green is also presented in Fig. 5.

Step 10: Use the approach for evaluation of the unknown exposure scenarios.

With the DCR-based predictions being evaluated, the use of the DCR approach for the safety evaluation of putative estrogenic exposures was supported and enabled the evaluation of the 11 exposure scenarios for which the corresponding in vivo estrogenic effects were unknown (purple circles). 10 out of these 11 exposure scenarios had a DCR ≤ 1 and 1 had a DCR > 1, indicating to be negative and positive for in vivo estrogenicity, respectively.

## Discussion

In the DCR approach, the EAR of an exposure scenario to a test compound (EAR_test_) is compared to the EAR of safe human exposure to a comparator compound (EAR_comparator_). A DCR value ≤ 1 indicates that the evaluated exposure to the test compound is expected to be safe. Van Tongeren et al. (2021) used an in vitro-based definition of the EAR_comparator_ with the BMCL_05_ as safe level of exposure to comparator compounds to evaluate putative anti-androgenic test compounds based on the AR-CALUX assay. The results obtained indicated that this NGRA strategy might be of use to also evaluate other biological endpoints for which in vitro bioassay results are available. In the current work, this DCR approach with in vitro assay-based EAR values was further developed using an in vitro-based EAR_comparator_ value defined for GEN to evaluate 41 human estrogenic exposure scenarios to 14 model compounds including endogenous hormones, phthalates, ethyl paraben, pesticides, bisphenol A, phytoestrogens, the mycotoxin zearalenone, and drugs. The in vitro data were derived from concentration-response curves obtained in the estrogenic in vitro MCF-7/Bos proliferation assay, T47D ER-CALUX assay, or U2OS ERα-CALUX assay (Wang et al. 2014). The DCRs of the 41 exposure scenarios for the 14 test compounds were calculated taking into account differences in in vitro and in vivo protein binding. The calculated DCR values of the test compounds were evaluated against actual knowledge on the corresponding occurrence of in vivo estrogenic effects at the respective level of exposure.

GEN was selected as the comparator compound because of (i) the wide range of available data on exposures that were reported to test negative for in vivo estrogenicity in humans and (ii) comparison of the free in vitro BMCL_05_ values to the reported free plasma concentrations at these non-estrogenic exposure levels. The fact that at the highest internal concentrations reported from supplement intake of GEN (Busby et al. 2002) no estrogenic effects were observed in the 30 male volunteers studied and that these concentrations were 13- to 34-fold higher than the free in vitro BMCL_05_ values of GEN (Fig. 3), provides additional support for the conclusion that exposure to the comparator GEN that results in an internal free concentration equal to the in vitro free BMCL_05_ can be considered safe and is adequate to calculate the EAR_comparator_ in the DCR approach. The large variation of the internal concentrations of GEN resulting from the different diets and within the different diets indicates that using human clinical or biomonitoring studies of GEN to define a safe level of exposure may leave substantial uncertainty. Furthermore, conflicting data on estrogenic (beneficial or adverse) effects are reported following GEN exposure. It is suggested that the effects can be dependent on, among others, sex, menstrual phase, and health status (Hargreaves et al. 1999; Khan et al. 2012; Niculescu et al. 2007; Petrakis et al. 1996; van der Velpen et al. 2014). Using reported internal concentrations of GEN to set the EAR_comparator_ values may therefore not be adequate. However, using in vitro*-*based BMCL_05_ values as an alternative safe level of exposure provides a more consistent way to set an adequate and safe EAR_comparator_. Thus, this novel in vitro-based EAR_comparator_ approach can be applied for endpoints for which a corresponding in vitro bioactivity assay is available, enabling the use of the DCR approach for many additional endpoints.

The use of this novel safe in vitro-based EAR_comparator_ in the DCR approach resulted in the correct prediction of the occurrence of in vivo estrogenic activity of the exposure scenarios for the various model compounds (Fig. 4), without the occurrence of false negatives, and, after reconsideration of the data for ZEA also without false positives. This further highlights that data from in vitro bioactivity assays are suitable for use in the DCR approach to evaluate the estrogenicity of compounds. The U2OS ERα-CALUX assay seems to provide the most conservative approach for setting DCR values for estrogenic exposure scenarios, generating relatively higher DCR values for the different exposure scenarios and thus being more likely to predict in vivo estrogenicity, than the approaches based on the T47D ER-CALUX assay and MCF-7/Bos proliferation assay. The MCF-7/Bos proliferation assay seemed the least conservative generating relatively lower DCR values for estrogenic exposure scenarios so that evaluation by this approach is less likely to predicted in vivo estrogenicity, thus easier suggesting a scenario to be safe. For all 3 approaches there was initially one false positive DCR outcome (Fig. 4), namely for the exposure to ZEA at a level below the established TDI (Fan et al. 2019). The reported PDIs of ZEA at the reported internal exposure levels evaluated in this scenario were lower than the TDI of ZEA of 0.25 µg/kg bw/day established based on the NOEL for estrogenic effects of ZEA and its metabolites on the ovary, uterus, and vulva in pigs (Alexander et al. 2011). However, this PDI was calculated using only kinetic parameters for urinary excretion and is thus a rough estimation rather than an exact assessment. Using a PBK model describing the ADME of ZEA in humans (Mendez-Catala et al. 2021) provided a more accurate prediction of the external dose. The PBK model-based prediction of the external doses at the internal exposure levels reported by Fan et al. (2019) were 3 to 4 orders of magnitude higher than the TDI of ZEA and the calculated PDIs of Fan et al. (2019). This indicates that these PBK model based calculations show that at the reported exposure there is a risk of in vivo estrogenicity and that the corresponding DCR values were thus correctly predicted by the DCR approach to be > 1. To further evaluate the DCR-based predictions of exposure to ZEA, the DCR at the EDI and TDI were calculated and were indeed < 1 (Fig. 5). The DCR-based safety decisions on the KEP exposure scenarios reported by Cannon et al. (1978) and Adir et al. (1978) were predicted based on the assumption of a male populations, which enabled comparison to the NOEL set in men based on a clinically relevant decrease in sperm count (Guzelian 1992). The DCR outcomes thus confirm that the assumption made was adequate.

The DCR predictions being validated enabled the safety estimation of the 11 exposure scenarios to model compounds for which it was unknown as to whether they would result in in vivo estrogenicity in humans (Fig. 4). Of these 11 exposure scenarios, 10 had a DCR ≤ 1 and 1 had a DCR > 1 and are thus expected to be negative and positive for in vivo estrogenicity, respectively.

To cover variability, EAR_test_ values of the test compounds used for the DCR analysis included, when available, lowest, mean, and highest EAR_test_ values calculated using lowest, mean, and highest internal dose levels of the exposure scenarios. The DCR obtained with the highest, or when not available the mean EAR_test_ values, was used to make a conservative safety decision on the exposure scenario to the respective test compound. As already stated, this approach correctly predicted the in vivo estrogenicity (Fig. 4). In this work, a cut-off of DCR ≤ 1 was used to estimate the estrogenicity of the studied exposure scenarios to the test compounds because the BMCL_05_ value reflecting an internal dose level without estrogenicity for the comparator compound GEN was considered safe and adequate to be used in the DCR approach. However, in future work, it can be considered whether in defining a cut-off for the DCR also uncertainty has to be taken into account, choosing a value lower than 1 for the cut-off since this will result in an even more conservative DCR-based safety decision.

When applying the NGRA approach based on in vitro studies it is important to note that the in vitro bioactivity assays that can be used in the DCR approach rarely capture toxicokinetics, such as metabolism, as in the human body (Coecke et al. 2006). BBzP, DBP, o,p’-DDT, ZEA, and TAM are known to be converted to more bioactive metabolites which will contribute to the in vivo estrogenicity of the respective parent compound. When using the three in vitro bioactivity assays in the DCR approach, this contribution to the estrogenicity may not be captured so that the observed in vitro toxicity of a parent compound may underestimate the toxicity in the human body. This issue can be overcome by using PBK models describing the kinetics of a parent compound and its respective relevant metabolites in humans enabling the prediction of the corresponding combined internal concentrations in parent compound equivalents (Mendez-Catala et al. 2021; van Tongeren et al. 2022; Wang et al. 2020). Furthermore, co-incubation with liver S9 fraction in the in vitro bioactivity assays (Mollergues et al. 2017) offers the opportunity to evaluate whether a compound will be converted to hepatic metabolites and whether they would be more potent to the corresponding biological target. Such strategies could be implemented in the DCR approach to overcome this limitation.

When exposure to a novel chemical is to be evaluated for estrogenic effects by the DCR approach, one may choose to use the most conservative assay, in this case the U2OS ERα-CALUX assay, instead of all three assays to reduce the labor intensity and use of resources. The endpoints of gene expression in the CALUX assays which are more upstream in the adverse outcome pathway (Legler et al. 1999; Sonneveld et al. 2005; van der Burg et al. 2010), may be more sensitive, and this may explain the more conservative evaluation compared to the DCR approach based on the more functional endpoint of estrogen-induced proliferation of the cells measured in the MCF/7-Bos proliferation assay (Soto et al. 1995). Furthermore, one may also choose to use the assay which is the least time consuming, which in this case are the CALUX assays with only a 24 h exposure time compared to the 6 days exposure time in the MCF/7-Bos proliferation assay. The results of the present study revealed that in principle all 3 bioassays resulted in similar outcomes. This is related to the fact that when using a less sensitive bioassay not only the EC_50_ values of the test compounds will be higher but also the EC_50_ value of the comparator will be higher, i.e., the relative potency of the compound is similar in all 3 bioassays, resulting in lower EAR_test_ and EAR_comparator_ values and thus comparable DCR values.

The present study focusing on estrogenicity and a previous study focusing on anti-androgenicity (van Tongeren et al. 2022) showed that the DCR approach can offer a relatively quick analysis on the safety of a defined exposure scenario regarding biological endpoints of which corresponding in vitro bioactivity assays are available. In NGRA, a tiered workflow could be followed when an exposure to a (novel) compound is to be evaluated. For instance, with in silico tools like the molecular initiating events (MIE) ATLAS, a prediction can be made if a chemical has affinity to bind and thus interact with a biological target based on its molecular structure (Allen et al. 2018). When a perturbation on a certain biological endpoint is expected, the use of an in vitro bioactivity assay covering this endpoint and using an adequate EAR_comparator_ will enable the determination of the corresponding DCR. When the DCR is ≤ 1, it can be suggested that the studied exposure scenario for the compound of interest does not raise a safety concern whereas when the DCR is > 1, this test compound should be prioritized for further testing. To conclude, the DCR approach was further developed using multiple in vitro bioactivity assays for estrogenicity as the biological endpoint as 3R compliant NAM in NGRA to evaluate the safety of estrogenic exposures in humans.

## Supplementary Information

Below is the link to the electronic supplementary material.

Supplementary file1 (DOCX 134 KB)

## Data Availability

Availability of data and materials Supplementary materials (supplementary tables).
