# Supplementary material for: Next generation risk assessment of human exposure to estrogens using safe comparator compound values based on in vitro bioactivity assays
Source: Arch Toxicol. 2023 Apr 22;97(6):1547–75. doi: 10.1007/s00204-023-03480-w (PMC10182946; doi:10.1007/s00204-023-03480-w)
Supplement: Supplementary file 1 — Supplementary file1 (DOCX 134 KB) [file 204_2023_3480_MOESM1_ESM.docx]

**Supplementary material**

**Next generation risk assessment of human exposure to estrogens using safe comparator compound values based on *in vitro* bioactivity assays**

Tessa C.A. van Tongeren^1*^, Si Wang^1^, Paul L. Carmichael^2^, Ivonne M.C.M. Rietjens^1^, Hequn Li^2^

^1^Division of Toxicology, Wageningen University and Research, 6700 EA, Wageningen, NL

^2^Unilever Safety and Environmental Assurance Centre, Sharnbrook, Bedfordshire MK44 1LQ, UK

^*^Correspondence:
Corresponding Author [tessa.vantongeren@wur.nl](mailto:tessa.vantongeren@wur.nl)

# Supplementary material S1. Negative exposure scenarios to GEN

**Supplementary Table S1.** The reported nominal and transformed free internal concentrations of GEN, using the f_ub_ *_in vivo_*, following intake of different diets which are indicated to be conservative and health protective in humans

| **Reference** | **Diet** | **Exposure scenario(s)** | **Plasma, serum, or blood concentrations reported** | **Nominal internal concentrations (µM)^1, 2^** | **F_ub_ *_in vivo_*** | **Free internal plasma  concentrations (µM)** |
| --- | --- | --- | --- | --- | --- | --- |
| (Busby et al., 2002) | Western diet | Isoflavone intake of < 10 mg/d | Plasma | 3.80E-02 ± 22E-02 (mean ± SD) | 0.07 | 2.76E-03 ± 1.60E-03 |
| (Metzner et al., 2009) | Western diet | A 14 day prohibition of soy foods and their derivatives prior to the study | Plasma | 5.10E-03 ± 6.00E-04 (mean ± SEM) |  | 3.70E-04 ± 4.35E-05 |
| (Grace et al., 2004) | Western diet | EPIC-Norfolk following information from a 7 day dietary diary | Serum | 4.64E-05 ± 3.00E-05 (mean ± SD) |  | 3.36E-06 ± 2.18E-06 |
| (Heald et al., 2006) | Western diet | Control men from a case-control study of prostate cancer | Serum | 1.00E-04 (median) (4.00E-05 - 1.40E-04 (IQR)) |  | 7.25E-06 (2.90E-06 - 1.02E-05) |
| (Setchell et al., 2003) | Western diet | Consumption a soy-deficient diet for 1 months prior | Serum | 1.10E-05 ± 3.00E-06 (mean ± SEM) |  | 7.98E-07 ± 2.18E-07 |
| (Yuan et al., 2012) | Asian diet | Polyphenol-free diet for 10 days prior to the study | Plasma | 1.74E-03 ± 1.36E-03 (mean ± SD) |  | 1.26E-04 ± 9.86E-05 |
| (Setchell et al., 2011) | Asian diet | Consumption of 250 mL soy milk 2 per day for 3.5 day, equivalent to 19 mg GEN per day | Plasma | 1.65E-03 ± 4.60E-05 (mean ± SD) |  | 1.20E-04 ± 3.34E-06 |
| (Arai et al., 2000) | Asian diet | Estimated dietary intake of 30.1 mg/day | Plasma | 9.23E-04 (median) (7.60E-05 - 7.40E-03 (range)) |  | 6.69E-05 (5.51E-06 -  5.37E-04) |
| (Iwasaki et al., 2008) | Asian diet | Calculated GEN intake of 21.7 (16.8-26.1) mg/d from dietary assessment with a food frequency questionnaire | Plasma | 1.60E-03 (median) (8.70E-04 - 2.80E-03 (IQR)) |  | 1.16E-04 (6.31E-05 -  2.03E-04) |
| (Shimazu et al., 2011) | Asian diet | Calculated GEN intake (not specified) from dietary assessment with a food frequency questionnaire | Plasma | 8.00E-04 (median) (3.30E-04 – 1.40E-03 (IQR)) |  | 5.80E-05 (2.39E-05 -  1.02E-04) |
| (Takashima et al., 2004) | Asian diet | Calculated GEN intake of 22.9 ± 15.9 mg/d from hospital diet | Serum | 1.20E-03 ± 1.26E-03 (mean ± SEM) |  | 8.70E-05 ± 9.14E-05 |
| (Ritchie et al., 2004) | Asian diet | A specific diet was followed over a 24 hour recording period | Plasma | 3.80E-04 ± 3.80E-05 (mean ± SD) |  | 2.76E-05 ± 2.76E-06 |
| (Busby et al., 2002), Formulation A | Supplement intake | Single oral dose of 8 mg/kg bw of a 90% GEN formulation | Plasma | 0.13 ± 0.02 (mean ± SD) |  | 9.50E-03 ± 1.52E-03 |
| (Busby et al., 2002), Formulation B | Supplement intake | Single oral dose of 8 mg/kg bw of a 45% GEN formulation | Plasma | 0.26 ± 0.16 (mean ± SD) |  | 1.89E-02 ± 1.16E-02 |
| (Metzner et al., 2009) | Supplement intake | Repeated dose of 30 mg GEN per day for 7 days | Plasma | 2.24E-02 ± 9.00E-03 (mean ± SEM) |  | 1.62E-03 ± 6.53E-04 |
| (Yuan et al., 2012) | Supplement intake | 4 mg GEN twice per day for 7 days | Plasma | 4.31E-03 ± 1.44E-03 (mean ± SD) |  | 3.13E-04 ± 1.04E-04 |
| (Setchell et al., 2003) (low dose) | Supplement intake | A single dose of 0.4 mg/kg bw | Serum | 4.95E-04 ± 8.10E-05 (mean ± SEM) |  | 3.59E-05 ± 5.87E-06 |
| (Setchell et al., 2003) (low dose repeat) | Supplement intake | Twice a dose of 0.4 mg/kg bw | Serum | 3.69E-04 ± 5.40E-05 (mean ± SEM) |  | 2.68E-05 ± 3.92E-06 |
| (Setchell et al., 2003) (low dose after food) | Supplement intake | A single dose of 0.4 mg/kg bw and another low dose after 1 week of daily soymilk consumption containing 50 mg natural isoflavones | Serum | 3.87E-04 ± 6.30E-05 (mean ± SEM) |  | 2.81E-05 ± 4.57E-06 |
| (Setchell et al., 2003) (moderate dose) | Supplement intake | A single dose of 0.8 mg/kg | Serum | 7.83E-04 ± 1.26E-04 (mean ± SEM) |  | 5.68E-05 ± 9.14E-06 |
| (Setchell et al., 2011) | Supplement intake | A single oral dose of 50 mg GEN | Plasma | 1.92E-02 ± 2.96E-03(mean ± SEM) |  | 1.39E-03 ± 2.15E-04 |

^1^ As derived from (Becker et al., 2015).

^2^ Serum concentration were assumed to be equal to plasma concentrations.

# Supplementary material S2. BMC analysis

The benchmark concentration (BMC) analysis of GEN was performed based on the *in vitro* concentration response data reported by Wang et al. (2014), using BMDS3.2.1 software (U.S. EPA). The benchmark response (BMR) was defined as a 5% extra response (BMR05). The BMC05 and its upper (BMCU05) and lower (BMCL05) 95% confidence interval were also determined. The model was accepted when the fitted model had a p-value > 0.01, a BMDU_05_: BMDL_05_ ratio (precision factor) below 3, or the lowest AIC, indicating support for a concentration response.

## Supplementary material S2.1 BMC modelling of *in vitro* concentration response data of GEN in the MCF-7/Bos proliferation assay

**Supplementary Table S2.1.1.** Input values of the *in vitro* concentration response data of GEN in the MCF-7/Bos proliferation assay (Wang et al., 2014)

| **Concentration (µM)** | **n** | **Response** | **SEM** |
| --- | --- | --- | --- |
| 0.006 | 3 | 0.05 | 0.02 |
| 0.02 | 3 | 0.17 | 0.02 |
| 0.06 | 3 | 0.35 | 0.02 |
| 0.2 | 3 | 0.55 | 0.04 |
| 0.6 | 3 | 0.64 | 0.02 |
| 2 | 3 | 0.63 | 0.08 |
| 6 | 3 | 0.54 | 0.06 |

**Supplementary Table S2.1.2.** BMC analysis of the *in vitro* concentration response data of GEN in the MCF-7/Bos proliferation assay (Wang et al., 2014). BMC_05_ and BMCL_05_ values were obtained using BMDS software version 3.2.1, at a BMC of 5% extra risk, BMR type Hybrid model-extra risk with normal distribution and constant variance.

| **Model** | **BMC (µM)** | **BMCL (µM)** | **BMCU (µM)** | **Test 4 P‑Value** | **AIC** | **Accepted** |
| --- | --- | --- | --- | --- | --- | --- |
| [Exponential 2 (CV - normal)](file:///C:\Users\tonge015\AppData\Local\Microsoft\Windows\INetCache\Content.MSO\CABDB153.xlsx#'freq-exp2-rest-opt1'!A1) | 4.31 | 2.66 | 33.91 | <0.0001 | -1.24 | No |
| [Exponential 3 (CV - normal)](file:///C:\Users\tonge015\AppData\Local\Microsoft\Windows\INetCache\Content.MSO\CABDB153.xlsx#'freq-exp3-rest-opt1'!A1) | 4.31 | 2.66 | 33.91 | <0.0001 | -1.24 | No |
| [Exponential 4 (CV - normal)](file:///C:\Users\tonge015\AppData\Local\Microsoft\Windows\INetCache\Content.MSO\CABDB153.xlsx#'freq-exp4-rest-opt1'!A1) | 0.06 | 0.05 | 0.10 | <0.0001 | -26.06 | No |
| [Exponential 5 (CV - normal)](file:///C:\Users\tonge015\AppData\Local\Microsoft\Windows\INetCache\Content.MSO\CABDB153.xlsx#'freq-exp5-rest-opt1'!A1) | 0.06 | 0.05 | 0.12 | <0.0001 | -26.06 | No |
| [Hill (CV - normal)](file:///C:\Users\tonge015\AppData\Local\Microsoft\Windows\INetCache\Content.MSO\CABDB153.xlsx#'freq-hil-rest-opt1'!A1) | 0.01 | 3.48E-03 | 0.02 | 0.01 | -57.35 | Yes |
| [Polynomial Degree 6 (CV - normal)](file:///C:\Users\tonge015\AppData\Local\Microsoft\Windows\INetCache\Content.MSO\CABDB153.xlsx#'freq-ply6-rest-opt1'!A1) | 3.42 | 1.87 | 19.43 | <0.0001 | -1.86 | No |
| [Polynomial Degree 5 (CV - normal)](file:///C:\Users\tonge015\AppData\Local\Microsoft\Windows\INetCache\Content.MSO\CABDB153.xlsx#'freq-ply5-rest-opt1'!A1) | 3.42 | 1.87 | 19.43 | <0.0001 | -1.86 | No |
| [Polynomial Degree 4 (CV - normal)](file:///C:\Users\tonge015\AppData\Local\Microsoft\Windows\INetCache\Content.MSO\CABDB153.xlsx#'freq-ply4-rest-opt1'!A1) | 3.42 | 1.87 | 19.43 | <0.0001 | -1.86 | No |
| [Polynomial Degree 3 (CV - normal)](file:///C:\Users\tonge015\AppData\Local\Microsoft\Windows\INetCache\Content.MSO\CABDB153.xlsx#'freq-ply3-rest-opt1'!A1) | 3.42 | 1.87 | 19.43 | <0.0001 | -1.86 | No |
| [Polynomial Degree 2 (CV - normal)](file:///C:\Users\tonge015\AppData\Local\Microsoft\Windows\INetCache\Content.MSO\CABDB153.xlsx#'freq-ply2-rest-opt1'!A1) | 3.42 | 1.87 | 19.43 | <0.0001 | -1.86 | No |
| [Power (CV - normal)](file:///C:\Users\tonge015\AppData\Local\Microsoft\Windows\INetCache\Content.MSO\CABDB153.xlsx#'freq-pow-rest-opt1'!A1) | 3.42 | 1.87 | 19.43 | <0.0001 | -1.86 | No |
| [Linear (CV - normal)](file:///C:\Users\tonge015\AppData\Local\Microsoft\Windows\INetCache\Content.MSO\CABDB153.xlsx#'freq-lin-unrest-opt1'!A1) | 3.42 | 1.87 | 19.43 | <0.0001 | -1.86 | Yes |

## Supplementary material S2.2 BMC modelling of *in vitro* concentration response data of GEN in the T47D ER-CALUX assay

**Supplementary Table S2.2.1.** Input values of the *in vitro* concentration response data of GEN in the T47D ER-CALUX assay (Wang et al., 2014)

| **Concentration (µM)** | **n** | **Response** | **SEM** |
| --- | --- | --- | --- |
| 0.0003 | 3 | -10.00 | 6.25 |
| 0.001 | 3 | -15.37 | 4.91 |
| 0.003 | 3 | -9.58 | 2.01 |
| 0.01 | 3 | 6.47 | 1.79 |
| 0.03 | 3 | 67.17 | 0.89 |
| 0.1 | 3 | 107.11 | 9.15 |
| 0.3 | 3 | 129.64 | 6.03 |
| 1 | 3 | 210.20 | 6.70 |

**Supplementary Table S2.2.2.** BMC analysis of the *in vitro* concentration response data of GEN in the T47D ER-CALUX assay (Wang et al. 2014). BMC_05_ and BMCL_05_ values were obtained using BMDS software version 3.2.1, at a BMC of 5% extra risk, BMR type Hybrid model-extra risk with normal distribution and constant variance.

| **Model** | **BMC (µM)** | **BMCL (µM)** | **BMCU (µM)** | **Test 4 P‑Value** | **AIC** | **Accepted** |
| --- | --- | --- | --- | --- | --- | --- |
| [Exponential 2 (CV - normal)](file:///C:\Users\tonge015\AppData\Local\Microsoft\Windows\INetCache\Content.MSO\CABDB153.xlsx#'freq-exp2-rest-opt1'!A1) | -9999 | 0.00 | Infinity | <0.0001 | 292.43 | No |
| [Exponential 3 (CV - normal)](file:///C:\Users\tonge015\AppData\Local\Microsoft\Windows\INetCache\Content.MSO\CABDB153.xlsx#'freq-exp3-rest-opt1'!A1) | -9999 | 0.00 | Infinity | <0.0001 | 294.43 | No |
| [Exponential 4 (CV - normal)](file:///C:\Users\tonge015\AppData\Local\Microsoft\Windows\INetCache\Content.MSO\CABDB153.xlsx#'freq-exp4-rest-opt1'!A1) | -9999 | 0.00 | Infinity | <0.0001 | 294.43 | No |
| [Exponential 5 (CV - normal)](file:///C:\Users\tonge015\AppData\Local\Microsoft\Windows\INetCache\Content.MSO\CABDB153.xlsx#'freq-exp5-rest-opt1'!A1) | -9999 | 0.00 | Infinity | <0.0001 | 296.43 | No |
| [Hill (CV - normal)](file:///C:\Users\tonge015\AppData\Local\Microsoft\Windows\INetCache\Content.MSO\CABDB153.xlsx#'freq-hil-rest-opt1'!A1) | 4.88E-03 | 3.29E-03 | 7.98E-03 | <0.0001 | 208.38 | Yes |
| [Polynomial Degree 6 (CV - normal)](file:///C:\Users\tonge015\AppData\Local\Microsoft\Windows\INetCache\Content.MSO\CABDB153.xlsx#'freq-ply6-rest-opt1'!A1) | 0.14 | 0.11 | 0.21 | <0.0001 | 249.67 | No |
| [Polynomial Degree 5 (CV - normal)](file:///C:\Users\tonge015\AppData\Local\Microsoft\Windows\INetCache\Content.MSO\CABDB153.xlsx#'freq-ply5-rest-opt1'!A1) | 0.14 | 0.11 | 0.21 | <0.0001 | 249.67 | No |
| [Polynomial Degree 4 (CV - normal)](file:///C:\Users\tonge015\AppData\Local\Microsoft\Windows\INetCache\Content.MSO\CABDB153.xlsx#'freq-ply4-rest-opt1'!A1) | 0.14 | 0.11 | 0.21 | <0.0001 | 249.67 | No |
| [Polynomial Degree 3 (CV - normal)](file:///C:\Users\tonge015\AppData\Local\Microsoft\Windows\INetCache\Content.MSO\CABDB153.xlsx#'freq-ply3-rest-opt1'!A1) | 0.14 | 0.11 | 0.21 | <0.0001 | 249.67 | No |
| [Polynomial Degree 2 (CV - normal)](file:///C:\Users\tonge015\AppData\Local\Microsoft\Windows\INetCache\Content.MSO\CABDB153.xlsx#'freq-ply2-rest-opt1'!A1) | 0.14 | 0.11 | 0.21 | <0.0001 | 249.67 | No |
| [Power (CV - normal)](file:///C:\Users\tonge015\AppData\Local\Microsoft\Windows\INetCache\Content.MSO\CABDB153.xlsx#'freq-pow-rest-opt1'!A1) | 0.14 | 0.11 | 0.21 | <0.0001 | 249.67 | No |
| [Linear (CV - normal)](file:///C:\Users\tonge015\AppData\Local\Microsoft\Windows\INetCache\Content.MSO\CABDB153.xlsx#'freq-lin-unrest-opt1'!A1) | 0.14 | 0.11 | 0.20 | <0.0001 | 253.51 | Yes |

## Supplementary material S2.3 BMC modelling of *in vitro* concentration response data of GEN in the U2OS ERα-CALUX assay

**Supplementary Table S2.3.1.** Input values of the *in vitro* concentration response data of GEN in the U2OS ERα-CALUX assay (Wang et al., 2014).

| **Concentration (µM)** | **n** | **Response** | **SEM** |
| --- | --- | --- | --- |
| 0.0003 | 3 | -12.10 | 0.00 |
| 0.001 | 3 | -9.73 | 0.00 |
| 0.003 | 3 | -0.75 | 6.13 |
| 0.01 | 3 | 34.64 | 19.10 |
| 0.03 | 3 | 90.08 | 23.35 |
| 0.1 | 3 | 102.83 | 4.25 |
| 0.3 | 3 | 108.74 | 16.98 |
| 1 | 3 | 170.78 | 24.53 |

**Supplementary Table S2.3.2.** BMC analysis of the *in vitro* concentration response data of GEN in the U2OS ERα-CALUX assay (Wang et al., 2014). BMC_05_ and BMCL_05_ values were obtained using BMDS software version 3.2.1, at a BMC of 5% extra risk, BMR type Hybrid model-extra risk with normal distribution and constant variance.

| **Model** | **BMC (µM)** | **BMCL (µM)** | **BMCU (µM)** | **Test 4 P‑Value** | **AIC** | **Accepted** |
| --- | --- | --- | --- | --- | --- | --- |
| [Exponential 2 (CV - normal)](file:///C:\Users\tonge015\AppData\Local\Microsoft\Windows\INetCache\Content.MSO\CABDB153.xlsx#'freq-exp2-rest-opt1'!A1) | -9999 | 0 | Infinity | <0.0001 | 287.18 | No |
| [Exponential 3 (CV - normal)](file:///C:\Users\tonge015\AppData\Local\Microsoft\Windows\INetCache\Content.MSO\CABDB153.xlsx#'freq-exp3-rest-opt1'!A1) | -9999 | 0 | Infinity | <0.0001 | 289.18 | No |
| [Exponential 4 (CV - normal)](file:///C:\Users\tonge015\AppData\Local\Microsoft\Windows\INetCache\Content.MSO\CABDB153.xlsx#'freq-exp4-rest-opt1'!A1) | -9999 | 0 | Infinity | <0.0001 | 289.18 | No |
| [Exponential 5 (CV - normal)](file:///C:\Users\tonge015\AppData\Local\Microsoft\Windows\INetCache\Content.MSO\CABDB153.xlsx#'freq-exp5-rest-opt1'!A1) | -9999 | 0 | Infinity | <0.0001 | 291.18 | No |
| [Hill (CV - normal)](file:///C:\Users\tonge015\AppData\Local\Microsoft\Windows\INetCache\Content.MSO\CABDB153.xlsx#'freq-hil-rest-opt1'!A1) | 2.34E-03 | 1.34E-03 | 4.83E-03 | 0.0005 | 219.11 | Yes |
| [Polynomial Degree 6 (CV - normal)](file:///C:\Users\tonge015\AppData\Local\Microsoft\Windows\INetCache\Content.MSO\CABDB153.xlsx#'freq-ply6-rest-opt1'!A1) | 0.20 | 0.15 | 0.34 | <0.0001 | 251.79 | No |
| [Polynomial Degree 5 (CV - normal)](file:///C:\Users\tonge015\AppData\Local\Microsoft\Windows\INetCache\Content.MSO\CABDB153.xlsx#'freq-ply5-rest-opt1'!A1) | 0.20 | 0.15 | 0.34 | <0.0001 | 251.79 | No |
| [Polynomial Degree 4 (CV - normal)](file:///C:\Users\tonge015\AppData\Local\Microsoft\Windows\INetCache\Content.MSO\CABDB153.xlsx#'freq-ply4-rest-opt1'!A1) | 0.20 | 0.15 | 0.34 | <0.0001 | 251.79 | No |
| [Polynomial Degree 3 (CV - normal)](file:///C:\Users\tonge015\AppData\Local\Microsoft\Windows\INetCache\Content.MSO\CABDB153.xlsx#'freq-ply3-rest-opt1'!A1) | 0.20 | 0.15 | 0.34 | <0.0001 | 251.79 | No |
| [Polynomial Degree 2 (CV - normal)](file:///C:\Users\tonge015\AppData\Local\Microsoft\Windows\INetCache\Content.MSO\CABDB153.xlsx#'freq-ply2-rest-opt1'!A1) | 0.20 | 0.15 | 0.34 | <0.0001 | 251.79 | No |
| [Power (CV - normal)](file:///C:\Users\tonge015\AppData\Local\Microsoft\Windows\INetCache\Content.MSO\CABDB153.xlsx#'freq-pow-rest-opt1'!A1) | 0.20 | 0.15 | 0.33 | <0.0001 | 251.79 | No |
| [Linear (CV - normal)](file:///C:\Users\tonge015\AppData\Local\Microsoft\Windows\INetCache\Content.MSO\CABDB153.xlsx#'freq-lin-unrest-opt1'!A1) | 0.19 | 0.15 | 0.29 | <0.0001 | 259.52 | Yes |

# Supplementary material S3. Evaluation of exposures to ZEA

**Supplementary Table S3.** The EAR_test_ values at the EDI and TDI of ZEA compounds based on the MCF-7/Bos proliferation assay, T47D ER-CALUX assay, and U2OS ERα-CALUX assay calculated using Eq. 2.

| **Compound** | **Exposure scenario(s)** | **Reference** | **EAR_test_ MCF-7/BOS proliferation** | | | **EAR_test_ T47D ER-CALUX** | | | **EAR_test_ U2OS ERα-CALUX** | | |
| --- | --- | --- | --- | --- | --- | --- | --- | --- | --- | --- | --- |
|  |  |  | **Lowest** | **Mean** | **Highest** | **Lowest** | **Mean** | **Highest** | **Lowest** | **Mean** | **Highest** |
| ZEA | TDI | Alexender et al., (2011) |  | 3.00E-04 |  |  | 1.96E-04 |  |  | 1.07E-04 |  |
| ZEA | EDI | Alexender et al., (2011) | 1.44E-06 | 2.78E-05 | 1.57E-04 | 9.38E-07 | 1.81E-05 | 1.02E-04 | 5.14E-07 | 9.94E-06 | 5.60E-05 |

**References**

Alexander, J. et al., (2011). Scientific Opinion on the risks for public health related to the presence of zearalenone in food. EFSA Journal, 9(6). <https://doi.org/10.2903/j.efsa.2011.2197>

Arai, Y., Uehara, M., Sato, Y., Kimira, M., Eboshida, A., Adlercreutz, H., & Watanabe, S. (2000). Comparison of isoflavones among dietary intake, plasma concentration and urinary excretion for accurate estimation of phytoestrogen intake. *Journal of Epidemiology*, *10*(2). https://doi.org/10.2188/jea.10.127

Becker, R. A., Friedman, K. P., Simon, T. W., Marty, M. S., Patlewicz, G., & Rowlands, J. C. (2015). An exposure: Activity profiling method for interpreting high-throughput screening data for estrogenic activity-Proof of concept. *Regulatory Toxicology and Pharmacology*, *71*(3). https://doi.org/10.1016/j.yrtph.2015.01.008

Busby, M. G., Jeffcoat, A. R., Bloedon, L. A. T., Koch, M. A., Black, T., Dix, K. J., Heizer, W. D., Thomas, B. F., Hill, J. M., Crowell, J. A., & Zeisel, S. H. (2002). Clinical characteristics and pharmacokinetics of purified soy isoflavones: Single-dose administration to healthy men. *American Journal of Clinical Nutrition*, *75*(1). https://doi.org/10.1093/ajcn/75.1.126

Grace, P. B., Taylor, J. I., Low, Y. L., Luben, R. N., Mulligan, A. A., Botting, N. P., Dowsett, M., Welch, A. A., Khaw, K. T., Wareham, N. J., Day, N. E., & Bingham, S. A. (2004). Phytoestrogen concentrations in serum and spot urine as biomarkers for dietary phytoestrogen intake and their relation to breast cancer risk in European Prospective Investigation of Cancer and Nutrition-Norfolk. *Cancer Epidemiology Biomarkers and Prevention*, *13*(5). https://doi.org/10.1158/1055-9965.698.13.5

Heald, C. L., Bolton-Smith, C., Ritchie, M. R., Morton, M. S., & Alexander, F. E. (2006). Phyto-oestrogen intake in Scottish men: Use of serum to validate a self-administered food-frequency questionnaire in older men. *European Journal of Clinical Nutrition*, *60*(1). https://doi.org/10.1038/sj.ejcn.1602277

Iwasaki, M., Inoue, M., Otani, T., Sasazuki, S., Kurahashi, N., Miura, T., Yamamoto, S., & Tsugane, S. (2008). Plasma isoflavone level and subsequent risk of breast cancer among japanese women: A nested case-control study from the Japan Public Health Center-based prospective study group. *Journal of Clinical Oncology*, *26*(10). https://doi.org/10.1200/JCO.2007.13.9964

Metzner, J. E., Frank, T., Kunz, I., Burger, D., & Riegger, C. (2009). Study on the pharmacokinetics of synthetic genistein after multiple oral intake in post-menopausal women. *Arzneimittel-Forschung/Drug Research*, *59*(10). https://doi.org/10.1055/s-0031-1296435

Ritchie, M. R., Morton, M. S., Deighton, N., Blake, A., & Cummings, J. H. (2004). Plasma and urinary phyto-oestrogens as biomarkers of intake: validation by duplicate diet analysis. *British Journal of Nutrition*, *91*(3). https://doi.org/10.1079/bjn20031062

Setchell, K. D. R., Brown, N. M., Zhao, X., Lindley, S. L., Heubi, J. E., King, E. C., & Messina, M. J. (2011). Soy isoflavone phase II metabolism differs between rodents and humans: Implications for the effect on breast cancer risk. *American Journal of Clinical Nutrition*, *94*(5). https://doi.org/10.3945/ajcn.111.019638

Setchell, K. D. R., Faughnan, M. S., Avades, T., Zimmer-Nechemias, L., Brown, N. M., Wolfe, B. E., Brashear, W. T., Desai, P., Oldfield, M. F., Botting, N. P., & Cassidy, A. (2003). Comparing the pharmacokinetics of daidzein and genistein with the use of 13C-labeled tracers in premenopausal women. *American Journal of Clinical Nutrition*, *77*(2). https://doi.org/10.1093/ajcn/77.2.411

Shimazu, T., Inoue, M., Sasazuki, S., Iwasaki, M., Sawada, N., Yamaji, T., & Tsugane, S. (2011). Plasma isoflavones and the risk of lung cancer in women: A nested case-control study in Japan. *Cancer Epidemiology Biomarkers and Prevention*, *20*(3). https://doi.org/10.1158/1055-9965.EPI-10-1025

Takashima, N., Miyanaga, N., Komiya, K., Mori, M., & Akaza, H. (2004). Blood isoflavone levels during intake of a controlled hospital diet. *Journal of Nutritional Science and Vitaminology*, *50*(4). https://doi.org/10.3177/jnsv.50.246

Wang, S., Aarts, J. M. M. J. G., de Haan, L. H. J., Argyriou, D., Peijnenburg, A. A. C. M., Rietjens, I. M. C. M., & Bovee, T. F. H. (2014). Towards an integrated in vitro strategy for estrogenicity testing. *Journal of Applied Toxicology*, *34*(9). https://doi.org/10.1002/jat.2928

Yuan, B., Zhen, H., Jin, Y., Xu, L., Jiang, X., Sun, S., Li, C., & Xu, H. (2012). Absorption and plasma disposition of genistin differ from those of genistein in healthy women. *Journal of Agricultural and Food Chemistry*, *60*(6). https://doi.org/10.1021/jf204421c
